# Afucosylation of HLA-specific IgG1 as a potential predictor of antibody pathogenicity in kidney transplantation

**DOI:** 10.1016/j.xcrm.2022.100818

**Published:** 2022-11-15

**Authors:** Pranay Bharadwaj, Sweta Shrestha, Tamas Pongracz, Catalano Concetta, Shilpee Sharma, Alain Le Moine, Noortje de Haan, Naoka Murakami, Leonardo V. Riella, Vanda Holovska, Manfred Wuhrer, Arnaud Marchant, Margaret E. Ackerman

**Affiliations:** 1 Department of Microbiology and Immunology, Geisel School of Medicine at Dartmouth, Dartmouth College, Hanover, NH 03755, USA; 2 Center for Proteomics and Metabolomics, Leiden University Medical Center, Leiden, the Netherlands; 3 Institute for Medical Immunology, Université Libre de Bruxelles, Charleroi, Belgium; 4 Department of Nephrology, Dialysis and Renal Transplantation, Hôpital Erasme, Université libre de Bruxelles, Bruxelles, Belgium; 5 Renal Division, Brigham and Women’s Hospital, Harvard Medical School, Boston, MA, USA; 6 Division of Nephrology, Massachusetts General Hospital, Harvard Medical School, Boston, MA, USA; 7 Center for Transplantation Sciences, Department of Surgery, Massachusetts General Hospital, Boston, MA, USA; 8 HLA Laboratory, Laboratoire Hospitalier Universitaire de Bruxelles (LHUB), Hôpital Erasme ULB, Brussels, Belgium; 9 Thayer School of Engineering, Dartmouth College, Hanover, NH 03755, USA

**Keywords:** IgG, effector function, donor-specific antibody, glycosylation, ADCC, antibody-mediated rejection, afucosylation, transplantation

## Abstract

Antibody-mediated rejection (AMR) is the leading cause of graft failure. While donor-specific antibodies (DSAs) are associated with a higher risk of AMR, not all patients with DSAs develop rejection, suggesting that the characteristics of alloantibodies determining their pathogenicity remain undefined. Using human leukocyte antigen (HLA)-A2-specific antibodies as a model, we apply systems serology tools to investigate qualitative features of immunoglobulin G (IgG) alloantibodies including Fc-glycosylation patterns and FcγR-binding properties. Levels of afucosylated anti-A2 antibodies are elevated in seropositive patients, especially those with AMR, suggesting potential cytotoxicity via FcγRIII-mediated mechanisms. Afucosylation of both glycoengineered monoclonal and naturally glycovariant polyclonal serum IgG specific to HLA-A2 drives potentiated binding to, slower dissociation from, and enhanced signaling through FcγRIII, a receptor widely expressed on innate effector cells, and greater cytotoxicity against HLA-A2^+^ cells mediated by natural killer (NK) cells. Collectively, these results suggest that afucosylated DSA may be a biomarker of AMR and contribute to pathogenesis.

## Highlights

• HLA-A2-specific antibodies are purified from reactive sera for Fc glycan analysis
• Afucosylated anti-A2 antibodies are elevated in sensitized subjects
• Afucosylated anti-A2 antibodies exhibit enhanced ADCC activity via FcγRIIIa
• Afucosylated DSA may be a biomarker of AMR and contribute to pathogenesis

## Graphical abstract

**Figure:**
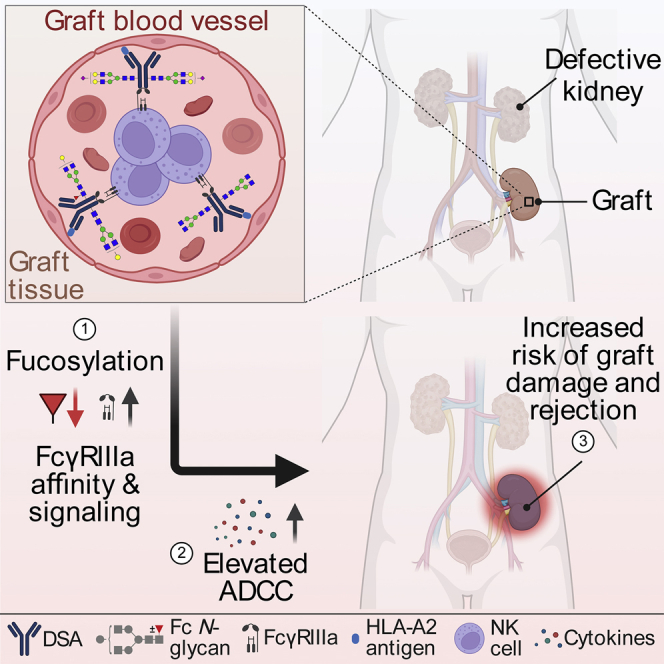

## Teaser

Bharadwaj et al. investigate relationships between Fc glycosylation profiles and antibody-mediated rejection (AMR) in kidney transplantation. They observe elevated afucosylated anti-A2 antibodies that are associated with cytotoxicity via FcγIII-mediated mechanisms, suggesting that afucosylated transplant-specific antibodies may be a biomarker and mechanism of AMR.

## Introduction

Antibody-mediated rejection (AMR), encompassing allograft rejection caused primarily by antibodies directed against donor-specific human leukocyte antigen (HLA) molecules, is the leading cause of solid organ transplant rejection and long-term graft loss.^1^^,^^2^^,^^3^^,^^4^^,^^5^ AMR is characterized by histological manifestations of endothelial cell injury, mononuclear cell infiltration, and complement-dependent tissue damage. Additionally, the presence of circulating donor-specific antibodies (DSAs) poses challenges for transplant recipients by limiting access to available organs, prolonging wait time, and, in some cases, excluding the candidates from a possible transplantation altogether.^6^^,^^7^^,^^8^ Solid phase assays using purified HLA antigens (Luminex single bead antigen assays) have significantly improved stratification and categorization of transplant candidates because they can detect very low levels of DSAs due to their high sensitivity. While this technique has been used to update organ allocation and desensitization protocols, it has led to minimal benefits to improvement in rejection treatment as the presence and levels of DSAs are not a reliable predictor of transplant outcome.^9^^,^^10^^,^^11^^,^^12^^,^^13^ While graft survival is clearly poorer for individuals sensitized against donor organ antigens,^1^^,^^2^^,^^14^^,^^15^^,^^16^^,^^17^ several studies have shown that not all DSAs carry the same risk of allograft rejection, as they have been associated with a wide spectrum of effects ranging from a complete absence of graft injury to the most severe form of AMR.^18^ Similarly, appearance of *de novo* DSAs implies the risk of graft deterioration but provides little to no information on their actual pathogenic activities.^3^^,^^19^^,^^20^^,^^21^^,^^22^

Collectively, these observations point to the importance of factors other than the magnitude of the DSA response as important drivers of pathology. Clinical practice is challenged by the lack of strong relationships between antibody characteristics and patient outcomes. Given the profound disproportion between available organs and the number of patients waiting for a transplant, the US Food and Drug Administration (FDA) recently identified AMR and desensitization as two important areas in transplantation for which no drugs have been specifically approved.^1^^,^^2^^,^^23^^,^^24^^,^^25^ Techniques that better define DSA properties may be required to first advance mechanistic understanding of AMR and to secondly improve transplant recipient outcomes.

To this end, the antibody effector functions, such as antibody-dependent cellular cytotoxicity (ADCC), complement-dependent cytotoxicity (CDC), phagocytosis, and induction of inflammatory response mediators that may be responsible for AMR are influenced not solely by titer but by affinity, antigen availability and epitope, and antibody isotype, subclass, and glycosylation.^12^^,^^26^^,^^27^^,^^28^^,^^29^^,^^30^^,^^31^ Among these traits, antibody Fc domain glycosylation is perhaps the least frequently characterized, despite relationships between the extent of fucosylation in modifying natural killer (NK)-cell-mediated cytotoxicity, galactose in modifying CDC, and sialylation having been associated with immunomodulatory effects.^32^^,^^33^^,^^34^^,^^35^^,^^36^^,^^37^ In other disease settings, systematic tools for surveillance of this spectrum of serum antibody features and their associated effector functions have identified reliable associations and begun to support robust predictions of disease outcomes.^38^^,^^39^^,^^40^^,^^41^^,^^42^^,^^43^^,^^44^^,^^45^ While prior research on the role of antibodies in transplant rejection was predominantly focused on estimation and retrospective correlation of titer to better predict transplant outcomes,^16^^,^^17^^,^^46^^,^^47^^,^^48^ recent studies have begun to interrogate other important factors governing antibody functionality, such as subclass distribution, complement fixing ability, and NK-cell-mediated cytotoxicity,^12^^,^^27^^,^^28^^,^^49^^,^^50^^,^^51^^,^^52^^,^^53^^,^^54^^,^^55^^,^^56^^,^^57^^,^^58^^,^^59^^,^^60^^,^^61^ and the evolution of these features with the progression of disease.^13^^,^^54^^,^^62^^,^^63^^,^^64^^,^^65^^,^^66^^,^^67^

Here, we report on the development and application of assays to characterize phenotypic and functional aspects of HLA-specific antibodies. Among a group of individuals with antibodies against HLA-A2, we observe altered Fc glycosylation profiles compared with total serum immunoglobulin G (IgG)—most notably, enrichment of afucosylated IgG1 antibodies, which are widely associated with potentiated ADCC.^35^^,^^68^^,^^69^^,^^70^^,^^71^^,^^72^ For both glycoengineered monoclonal and polyclonal human serum-derived HLA-A2-specific antibodies, binding to and signaling via FcγRIIIa, the receptor expressed on NK and other innate immune cells and responsible for mediating ADCC, were negatively associated with fucosylation. Collectively, this work establishes the potential importance of DSA Fc glycosylation in influencing the ADCC activity of DSA and, in turn, their ability to contribute to graft rejection.

## Results

HLA-specific antibody responses were investigated in a cohort consisting of individuals with reported clinical HLA-A2 reactivity (n = 32) as detected with a single bead antigen assay and controls (n = 18) with no HLA-A2 reactivity. Among anti-A2-sensitized patients, 28 had received an organ transplant, including 26 kidney transplants and 2 other organs, and 4 patients were on a transplant waiting list (Table S1). A2 sensitization was related to previous or current transplantation in 13 patients, pregnancy in six, blood transfusion in one, and left ventricular assistance device in one, whereas the sensitizing event was unknown in 4 patients. Thirteen out of the 28 (46%) patients with a transplant (Table S1) and 7 out of the 13 (54%) patients who were DSA positive (A2 sensitization in a patient transplanted with an HLA-A2 graft) (Table S2) were diagnosed with either acute or chronic AMR. Among controls, nine patients had no detectable anti-HLA antibodies, and nine had detectable antibodies against non-HLA-A2 antigens. Seventeen controls had received an organ transplant, and one was on a transplant waiting list.

### IgG subclass distribution of HLA-specific antibodies

**Figure 1:**
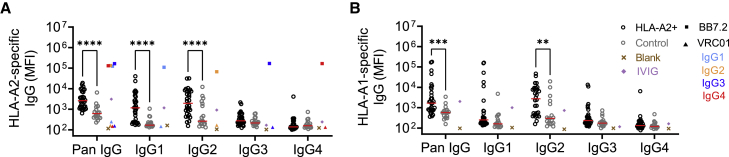
Subclass distribution of HLA-specific antibodies Characterization of HLA-specific antibodies binding to HLA-A2 (A) and HLA-A1 (B) antigens across IgG subclasses in HLA-A2-positive (hollow black circles; n = 30–32) and control (hollow gray circles; n = 18) individuals. HLA-A2-specific BB7.2 (square) and HIV-specific VRC01 (triangle) mAb subclass controls for HLA-A2 reactivity are shown for each subclass: IgG1 (light blue), IgG2 (orange), IgG3 (dark blue), and IgG4 (red). Buffer only blank (cross) and pooled IVIG (diamond) are shown in brown and purple, respectively. Serum samples were tested at a 1:100 dilution. Data shown are representative of two technical replicates. Solid red lines indicate group median. Differences between groups were evaluated using ordinary two-way ANOVA adjusted for multiple comparisons using Bonferroni’s test (∗∗p < 0.01, ∗∗∗p < 0.001, and ∗∗∗∗p < 0.0001, respectively).

We first sought to characterize the subclasses of HLA-specific IgGs in HLA-A2-sensitized and control subjects, using research-grade single-antigen bead immunoassays. Briefly, single antigens (intact HLA-A∗02:01 or HLA-A∗01:01 monomers) were captured on microspheres, and antigen binding of sera samples was measured by multiplex assay. In contrast to the HIV-specific antibody VRC01 (negative control), the HLA-A2-specific monoclonal antibody BB7.2 (positive control), which was evaluated in each human IgG subclass, showed specific binding to HLA-A2 (Figure 1A) and appropriate staining by IgG subclass detection reagents (Figure S1). In contrast to the negative control monoclonal antibody (mAb), both pooled human serum Ig (IVIG) and serum from individual control subjects often showed signal considerably above background. While this profile may be consistent with the widespread prevalence of anti-HLA antibodies among healthy individuals,^73^^,^^74^ and responses among subjects clinically defined as HLA-A2 seropositive were significantly elevated compared with controls, there was a considerable overlap in distributions. Nonetheless, there was a distinct difference observed in the subclass distribution between the two groups, with the HLA-A2-seropositive group generally exhibiting a wide range of total HLA-A2 antibodies compared with the control samples, which tended to show lower levels (Figure 1A). The wide distribution of the HLA-A2-specific IgG signal was consistent with reported values from clinical testing (Figure S2), with some samples exhibiting moderate to very low HLA-A2 reactivity. HLA-A2-reactive antibodies predominantly belonged to the IgG1 and IgG2 subclasses, but a few subjects also exhibited elevated levels of IgG3 and IgG4; these individuals typically had high levels of total HLA-A2-specific IgG.

A similar analysis of HLA-A1-specific antibody levels was performed. The HLA-A2-seropositive group also showed elevated levels of HLA-A1 antibodies compared with the control group (Figure 1B), again, consistent with clinical single-antigen bead assay results. Interestingly, HLA-A1-specific antibodies predominantly belonged to the IgG2 subclass, with some samples exhibiting higher levels of IgG1, IgG3, and IgG4 subclasses.

### Affinity enrichment yields HLA-A2-specific antibodies

A microscale purification method was successfully developed and employed for the purification of a control murine HLA-A2-specific mAb spiked into IVIG (Figure S3). Purified antibodies showed elevated reactivity to HLA-A2 antigen, but not to HLA-A1 or to a herpes simplex virus (HSV) antigen, which were used as negative controls, confirming the purity of the antibodies. Having vetted the purification process, HLA-A2-specific antibodies were then purified from serum of HLA-A2-antibody-seropositive patients. The purified antibody fractions showed elevated reactivity to HLA-A2 but not to HSV. Despite generally being thought not to possess shared epitopes, the HLA-A2-enriched fraction showed some cross-reactivity with HLA-A1 for a few subjects who were generally strongly positive for both HLA-A1 and HLA-A2 antibodies (based on clinical single-antigen bead assay) (Figure S3).

### HLA-A2-specific antibodies exhibit variable degree of afucosylation

**Figure 2:**
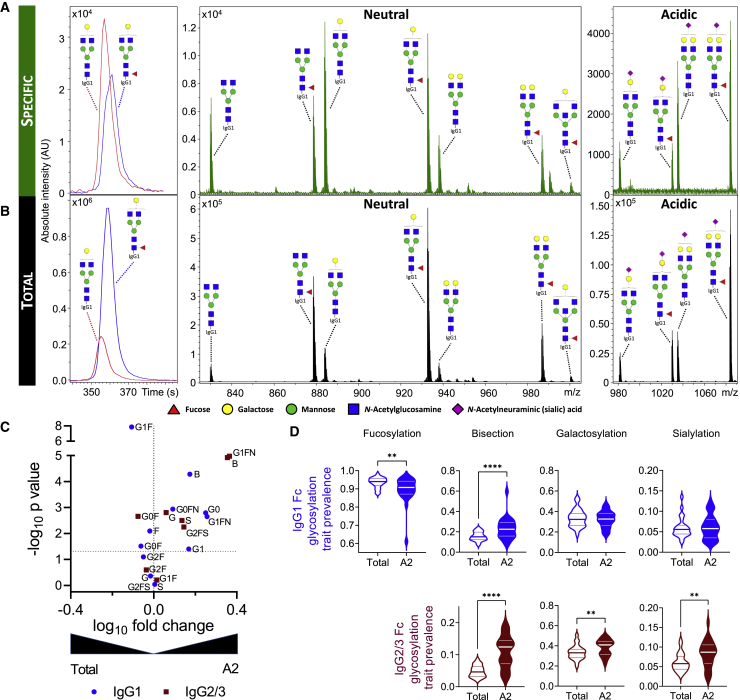
Fc glycosylation of HLA-A2-specific antibodies (A and B) Representative extracted ion chromatograms and mass spectra illustrating the observed variability between HLA-A2-specific (A) and total (B) IgG1 Fc glycosylation patterns of the same patient. (C) Volcano plot displaying the log10-fold change (x axis) and −log10 p value (y axis) of individual IgG1 (blue circle) and IgG2/3 (maroon square) glycoforms between total and HLA-A2-specific IgG1 and IgG2/3, respectively. (D) Violin plots showing the relative prevalence of glycans on bulk and HLA-A2-specific IgG1 (top) and IgG2/3 (bottom), respectively. Statistical analysis was performed using a paired two-tailed Student’s t test (∗∗p < 0.01, ∗∗∗p < 0.001, and ∗∗∗∗p < 0.0001, respectively).

Having confirmed the largely selective enrichment of HLA-A2-reactive antibodies, next we analyzed Fc glycosylation of both HLA-A2-specific and total serum IgG among HLA-A2-seropositive individuals. Fc glycosylation analysis revealed differences both on the level of individual glycoforms (Figures 2A–2C) and overall glycosylation traits including levels of fucosylation, bisection, galactosylation, and sialyation (Figure 2D) between HLA-A2-specific and total serum IgG1. Of note, while HLA-A2-specific antibody Fc fucosylation of some individuals was comparable to that of total IgG1, others exhibited fucosylation levels reduced by 5%–15%, or even 30%, compared with their total IgG1 counterpart (Figure 2D). HLA-A2-specific IgG2/3 was characterized by higher bisection, galactosylation, and sialylation than total serum IgG2/3, but no afucosylated IgG2/3 glycopeptides were detected (Figure 2D). Consistent with multiplex assay data, antigen-specific IgG4 responses were generally too low to be robustly characterized by liquid chromatography with mass spectrometry (LC-MS). While IgG2 and IgG3 glycopeptides could not be distinguished by MS due to their identical molecular mass, the high levels of antigen-specific IgG2 and low levels of IgG3 observed suggest that the antigen-specific IgG2/3 glycosylation signatures were dominated by IgG2.

### Afucosylated HLA-A2-specific mAbs exhibit enhanced FcγRIIIa signaling and ADCC activity

**Figure 3:**
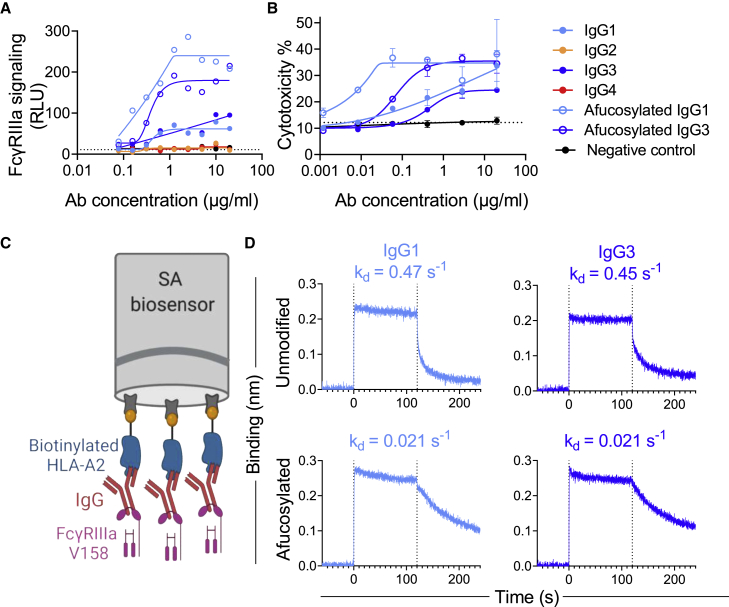
Impact of HLA-A2-specific mAb fucosylation on FcγRIIIa binding, signaling, and cytotoxic activity per subclass (A) FcγRIIIa signaling in a reporter cell line assay with unmodified and afucosylated HLA-A2 mAbs of varying subclasses. (B) Death (percentage of cytotoxicity) of HLA-A2+ target cells following coculture with NK cells in the presence or absence of antibodies of varying specificity, subclass, and fucosylation status. Connecting lines indicate curve fit models. Error bars indicate mean and SD of duplicates. Dotted horizontal line represents ADCC activity in the absence of antibody. (C) Schematic figure of the BLI experiment to define FcγRIIIa off rates from HLA-A2-specific antibodies. Illustration created with http://BioRender.com. (D) FcγRIIIa V158 association with and dissociation from unmodified and afucosylated HLA-A2 mAbs. Dissociation rate (K_D_) values are shown in inset.

To study the FcγR signaling characteristics of HLA-A2-specific antibodies, we produced afucosylated BB7.2 IgG1 and IgG3 mAbs and assessed signaling driven by ligation of FcγRIII, a receptor typically expressed by NK cells, in a reporter cell line^75^ as a surrogate for ADCC activity. IgG1 and IgG3 isotypes of BB7.2 were found to show FcγRIIIa ligation and signaling activity, while neither the control IgG1 subclass nor the IgG2 and IgG4 subclasses of BB7.2 showed activity above baseline (Figure 3A). As expected, afucosylated IgG1 and IgG3 demonstrated considerably enhanced FcγRIII signaling activity compared with their non-glycoengineered counterparts.

Lastly, we evaluated the cytotoxic activity of HLA-A2-specific antibodies by measuring the antibody-dependent killing of HLA-A2+ A375 cells, a human melanoma cell line, mediated by NK-92, a human NK cell line. Similar to the FcγRIIIa signaling activity, afucosylated IgG1 and IgG3 demonstrated higher ADCC activity compared with their non-glycoengineered counterparts and a negative control antibody across a range of antibody concentrations (Figure 3B).

### Enhanced FcγRIII signaling and ADCC activity are associated with slow antibody dissociation

To address the mechanism of improved FcγRIIIa signaling and ADCC activity, we next sought to characterize the interaction between HLA-A2-specific antibodies and FcγRIIIa using biolayer interferometry (BLI) (Figures 3C and S4), a label-free technique commonly used to study the affinity and kinetics of protein-protein interactions. Antibodies bound to HLA-A2 antigen were allowed to bind (t = 0–120 s) and then dissociate (t = 120–240 s) from FcγRIIIa V158. All mAbs exhibited the fast on rate typical of FcγR and showed similar levels of FcγRIIIa-binding signal (Figure 3D). In contrast, dissociation rates (K_D_) varied by more than an order of magnitude between fucosylated and afucosylated mAbs (Figure 3D). We found that afucosylated IgG1 and IgG3 had slower dissociation rates (mean K_D_ = 0.021 s^−1^) compared with unmodified forms (mean K_D_ = 0.46 s^−1^). This difference in dissociation rate was specific for FcγRIIIa as fucosylation did not influence FcγRI binding (Figure S4), as expected.^35^

### Serum HLA-A2-specific antibody fucosylation associates with FcγRIIIa dissociation and ligation

**Figure 4:**
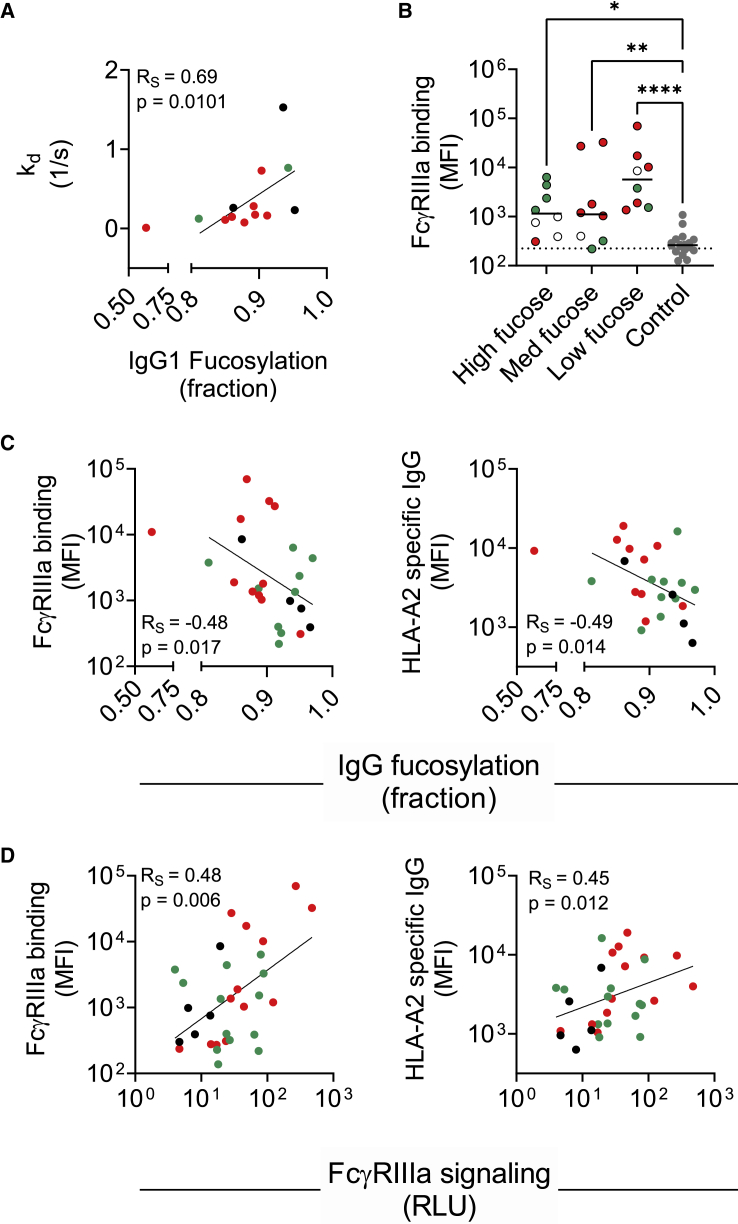
Associations of serum-derived HLA-A2-specific antibody fucosylation with FcγRIIIa binding and signaling (A) Spearman’s correlation (R_S_) between IgG1 fucosylation and FcγRIIIa dissociation rate (n = 13). (B) FcγRIIIa binding characterization in high (n = 8), medium (n = 8), and low (n = 8) fucose samples and negative controls (n = 18). Serum samples were tested at a 1:500 serum dilution. Statistical analysis was performed using ordinary one-way ANOVA adjusted for multiple comparisons using Tukey’s test. Solid lines indicate group median. Data shown are representative of two technical replicates. (C and D) Spearman’s correlations between IgG1 fucosylation (n = 24) (C) and FcγRIIIa signaling (n = 31) (D) with FcγRIIIa binding (left) and HLA-A2-specific IgG levels (mean fluorescence intensity [MFI]) (right). Patients with AMR (red), patients without AMR (green), and patients with no AMR information (black) are indicated in color.

Since afucosylated HLA-A2-specific mAbs had slower dissociation from FcγRIIIa compared with their unmodified counterparts, we next studied the dissociation profiles of polyclonal HLA-A2-specific antibodies in human serum and found the FcγRIIIa dissociation rate to be positively associated with fucosylation (Figure 4A). The variable off rates observed among differentially fucosylated serum-derived HLA-A2-specific antibodies suggested that FcγRIIIa ligation and signaling profiles of these HLA-A2-specific antibodies might also vary. To test this possibility, HLA-A2-seropositive subjects were split into tertiles (high, medium, and low) based on their HLA-A2-specific antibody fucosylation levels (Figure 4B). A multiplex assay was conducted in which HLA-A2-specific antibodies were evaluated for their ability to bind FcγRIIIa tetramers.^76^ Although there was no significant difference in FcγRIIIa binding between high, medium, and low fucose tertiles, HLA-A2-specific antibodies in each of these three categories had higher FcγRIIIa binding compared with negative controls, as exemplified by the observed negative correlations (Figure 4B). In line with observations on variably fucosylated mAbs, these results demonstrate that human serum-derived HLA-A2-specific antibodies with low fucosylation exhibit improved FcγRIIIa binding.

### Serum HLA-A2-specific antibody fucosylation associates with functional activity

To understand how antibody fucosylation level was related to FcγRIIIa signaling, a limited correlation analysis was performed. Both HLA-A2-specific antibody response magnitude and the FcγRIIIa ligation activity determined by multiplex assay were negatively associated with fucosylation (Figure 4C) and positively associated with FcγRIIIa signaling (Figure 4D). Despite the low signaling activity observed across experimental replicates in the reporter cell line assay (Figure S5A), measured relationships between FcγRIIIa signaling were consistently correlated with polyclonal HLA-A2-specific antibody response magnitude and FcγRIIIa binding (Figure S5B).

### HLA-A2-specific antibody afucosylation was correlated with clinical AMR

**Figure 5:**
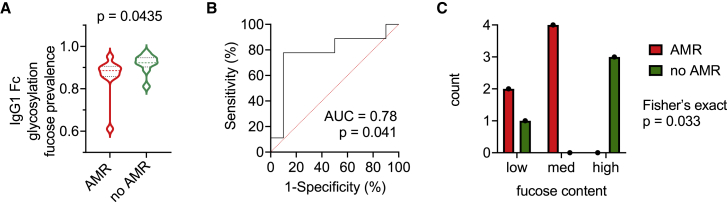
HLA-A2-specific IgG1 afucosylation is associated with AMR (A) Violin plot showing relative prevalence of fucose on HLA-A2-specific IgG1 in individuals with AMR (n = 10) and without AMR (n = 9). A Mann-Whitney U test was used to compare the two groups. (B) Receiver operating characteristic (ROC) curve and area under the curve (AUC) depicting performance of AMR status classification across increasing IgG1 fucose prevalence thresholds. (C) Number of subjects in which HLA-A2-specific IgG1 comprise a DSA plotted by fucose content as tertiles (low, medium, high) and AMR status. Statistical significance defined by Fisher’s exact test.

To explore how HLA-A2-specific antibody characteristics relate to transplantation outcomes, patient data were analyzed based on AMR status. Subjects with AMR tended to have high HLA-A2 antibody responses and low HLA-A2-specific IgG1 fucosylation, thereby showing high FcγRIIIa binding, high signaling activity, and slower dissociation from FcγRIIIa (Figure 4). As a more direct test, we analyzed HLA-A2-specific antibody features between subjects with and without AMR. Whether or not anti-HLA-A2 represented a DSA, individuals with AMR showed significantly lower levels of HLA-A2-specific IgG1 fucosylation compared with the individuals who did not have clinically defined AMR (Figure 5A). Receiver operating characteristic (ROC) curve analysis performed in the anti-A2-sensitized cohort (Figure 5B) to define the accuracy of AMR status classification based on IgG1 fucose prevalence achieved an area under the curve (AUC) of 0.78, indicating a good discrimination for individuals with different AMR outcomes, suggesting that a low level of HLA-A2-specific antibody fucosylation may be a marker of AMR in patients who have had a kidney transplant. Other features, including HLA-A2-specific antibody response magnitude, did not show such differences according to AMR status (Figures S5C–S5F).

In order to further examine the correlation between afucosylation of DSAs and clinical AMR outcomes, we analyzed subjects who had anti-HLA-A2 as a DSA. Patients who developed AMR had “low” and “moderate” fucose content of HLA-A2-specific DSAs, whereas patients who did not develop AMR had “high” fucose content of HLA-A2-specific DSAs (Figure 5C). Collectively, these data suggest that the phenotypic variability among anti-HLA-A2 antibody responses may have an important influence on organ transplant outcomes.

## Discussion

The last two decades have witnessed a considerable increase in understanding immunological processes related to organ rejection, which has contributed to reduction in the incidence of acute rejection and improvement in short-term graft survival.^23^^,^^77^^,^^78^^,^^79^ Despite the advances in immunosuppression strategies, HLA matching, and better management of acute rejection, the prognosis for long-term graft outcome remains one of the biggest challenges in the field of organ transplantation.^2^ Given the well-established importance of qualitative aspects of the antibody response in other settings, consideration of antibody subclass, Fc glycosylation, and FcγR binding in the context of renal transplant outcomes will define the importance of these features in mediating functions that escalate the risk of organ rejection.^2^ Discovery of robust relationships between features of DSAs and transplant outcomes has the potential to drive revision of clinical strategies of organ allocation as well as development of novel interventions.

To date, relatively few studies have looked at DSA subclass and Fc glycosylation in kidney transplant recipients. Challenges in DSA purification from plasma/serum appear to have posed a major hurdle to its glycosylation analysis, resulting in a focus on characterization of total serum IgG glycosylation.^80^^,^^81^^,^^82^ In this study, we employed a microscale purification method to purify HLA-A2-specific antibodies from patient sera and set out to evaluate IgG subclass and Fc glycosylation profiles thereof. Both of these features regulate Fc-FcγR interactions, which in turn potentially relate to pathogenicity and graft rejection.

Beyond the complexity of the many processes associated with allograft injury and rejection, phenotypic changes in DSA over time have also been observed,^49^ complicating the understanding of the pathophysiology of rejection. What has become clear is that DSA titers, subclass profiles, or C1q binding activity alone are insufficient to accurately predict the course of AMR, and the field remains in need of reliable prognostic biomarkers to guide treatment, optimally allocate organs, and reduce the risk of rejection in DSA-sensitized recipients.

Though not strongly associated with outcomes of organ rejection, IgG subclass is known to strongly modify the ability of antibodies to drive complement deposition and recruit the innate immune effector cells that mediate clearance of opsonized particles.^5^^,^^27^^,^^29^^,^^49^^,^^83^ HLA-A2-specific responses in clinically seropositive subjects were predominantly IgG1 and IgG2. High levels of IgG1 antibodies suggest the possibility of elevated Fc-mediated effector functions, such as phagocytosis, ADCC, and complement deposition,^35^ which might lead to severe graft injury and transplant rejection. The prevalence of HLA-A1-specific IgG2 antibodies in this population suggests that the IgG subclass profile can vary from one HLA antigen to another even within a given individual, highlighting the potential importance of integrating profiles across diverse specificities and characterizing features of anti-HLA antibodies beyond titer.

Antibody effector functions are highly affected by the composition of the *N*-glycan present at the conserved *N*-glycosylation site in the Fc region.^32^^,^^70^^,^^71^^,^^84^^,^^85^^,^^86^ Hitherto, it has been repeatedly shown that disease-specific antibodies can exhibit skewed glycosylation profiles, which in turn associate with disease prognosis and outcome.^87^^,^^88^^,^^89^^,^^90^^,^^91^^,^^92^^,^^93^ Historically, one of the key limitations of glyco-profiling such antibodies is their low serum prevalence and high sample requirement. In order to facilitate Fc glycosylation analysis of low abundant HLA-A2-specific antibodies, an antigen-specific antibody purification approach was developed for reliable, sensitive, and specific capturing of HLA-A2-specific antibodies from reactive sera. This platform was leveraged to support analysis of HLA-A2-specific IgG Fc glycosylation profiles. Compared with the global serum IgG profiles, we observed variable degree of HLA-A2-specific IgG1s afucosylation. Altered fucosylation was observed across seropositive patients, whether or not HLA-A2 specificity represented a DSA, a potentially cross-reactive response to the graft, or may have been derived from antigenic exposure(s) unrelated to transplant. Definition as to whether cross-reactive antibodies or antibodies that are specific to distinct donor organ antigens exhibit similar glycoprofiles remains to be determined but has been observed in other disease contexts.^88^^,^^89^ This feature was associated with improved FcγRIIIa binding as afucosylation has been described to lead to elevated FcγRIII binding.^58^^,^^63^^,^^83^^,^^84^^,^^85^^,^^86^ Additionally, studies have shown that individuals with AMR exhibit higher expression of FCGR3A in NK cells and CD16a-inducible NK cell-selective transcripts compared with individuals without AMR,^58^^,^^59^^,^^61^^,^^94^ suggesting that the combination of enriched afucosylated antibodies and elevated FcγRIIIa expression could synergize, leading ADCC to be a key mechanism in graft injury. Hence, we hypothesized that low HLA-A2-specific IgG1 fucosylation could cause elevated DSA cytotoxicity via enhanced ADCC. Indeed, when tested in the context of a subclass-switched recombinant antibody specific for HLA-A2, both subclass and fucosylation had a strong impact on FcγRIIIa affinity and signaling. Specifically, afucosylated mAbs had slower dissociation from the receptor, which could provide sufficient time for the receptors to cross-link and activate downstream signaling pathways and modulate ADCC. This relationship was also observed among polyclonal HLA-A2-specific antibodies purified from patient sera and suggests that afucosylated antibodies to HLA-A2 can exhibit elevated ADCC activity, as shown in other disease settings,^44^^,^^89^^,^^95^^,^^96^^,^^97^ and raises the possibility that DSA glycosylation may provide prognostic value in predicting risk of AMR. Indeed, individuals who had AMR exhibited low levels of HLA-A2-specific IgG1 fucosylation compared with the individuals who did not have AMR, suggesting that low HLA-A2 IgG1 fucosylation may be a marker of AMR risk in patients who have had a kidney transplant. Despite the small cohort size, among individuals in whom HLA-A2 represented a DSA, low HLA-A2-specific IgG1 antibody fucosylation was associated with AMR.

While this work suggests the potential role of IgG Fc fucosylation and its influence on DSA pathogenicity, it is likely that more systematic serology data, considering antibody response magnitude, subclass, glycans, epitope specificity, antigen specificity, and affinity, linked to clinical metadata and insights into host- and graft-specific genetic and phenotypic factors will be needed for comprehensive understanding. Understanding the effect of IgG Fc glycosylation on other antibody effector functions such as complement activation is also of utmost interest, as complement activation has shown to play a major role in AMR,^50^^,^^52^ and recent studies have highlighted the importance of Fc galactosylation in enhancing classical complement activation.^32^^,^^98^ The experimental tools explored here will need to be broadened to cover the diversity of DSA specificities that contribute to clinical outcomes. Similarly, analytical approaches^99^^,^^100^^,^^101^ that integrate across features of the humoral response and that can robustly address correlations among features, such as those observed here between response magnitude and afucosylation, may contribute to the discovery of mechanisms underlying graft injury and AMR. To this end, the subclass-switched and glycoengineered mAbs described herein may support dissection of the mechanistic role of these features in AMR studies in animal models.

Overall, despite considerable clinical heterogeneity of the study population, HLA-A2-specific IgG fucosylation was associated with FcγRIIIa binding and function. Different HLA reactivities showed distinct subclass profiles, and the dominant IgG1 fraction of HLA-A2-specific IgG showed reduced fucosylation compared with total serum IgG1. Along with antibody response magnitude, reduced fucosylation was associated with elevated binding to, and slower dissociation from, FcγRIIIa. Afucosylation of HLA-A2-specific antibodies was also associated with potentiated signaling and NK-cell-mediated cytotoxicity through FcγRIIIa. HLA-A2-specific IgG1 Fc fucose content was associated with AMR status among the subset of subjects for whom this specificity represented a DSA, as well as among those where it did not, leaving open questions about the importance of this antibody feature in mediating AMR. Further and prospective studies will be needed to confirm the role of afucosylation in the pathophysiology of AMR, and continued customization and broader deployment of these and other systems serology approaches have the potential to shed light on the clinical impact of qualitative antibody features and activities on graft damage.

### Limitations of the study

Limitations of this study include its focus on single antigen specificity and antibody function, limited outcome data, and reliance on a relatively small group of HLA-A2-seropositive subjects, sampled at a single time point long after transplant that was variably timed with respect to DSA generation and AMR diagnosis. The lack of a clear boundary between seropositive and seronegative subjects based on clinical testing suggests that positivity determinations may be quite sensitive to different reagents, tests, and thresholds, as well as to differences in antigen density and loaded antigens between single-antigen beads and research microspheres applied by different clinical groups. Antibody clonality, fine epitope specificity, and affinity for HLA-A2 were not assessed. Despite extensive condition testing, signals in the reporter cell line assay were low for both mAbs and polyclonal sera samples and, despite their correlations with other activities, were not always elevated compared with the control sera. Similarly, while signals in the in-house multiplex assay were correlated with clinical test results, agreement was less good than what we have observed in other multiplexed assays, and distributions overlapped with those observed among control sera, perhaps in association with the rate of seropositivity in healthy populations.^73^^,^^74^ While similar yields were observed to result from affinity purification against HLA-A2 with each of two different peptides in a pilot experiment, peptide occupancy and identity may also impact results of the assays used. Antibody glycosylation can be somewhat dynamic^102^^,^^103^ but was tested at a single time point. Lastly, while a direct association has been made between afucosylation of DSAs and the occurrence of AMR, HLA-A2-specific antibodies were first detected in most of the patients post-rejection, suggesting that afucosylation of DSAs may have occurred as a result of AMR. Future studies will benefit from larger and longitudinal cohorts that include samples collected pre-AMR and that are more homogeneous in relevant clinical characteristics and designs that are targeted to address relationships with transplant outcomes.

## STAR★Methods

### Key resources table

**Table:** 

REAGENT or RESOURCE	SOURCE	IDENTIFIER
**Antibodies**		

Mouse Anti-Human IgG Fc-PE (JDC-10)	SouthernBiotech	Cat# 9040-09; RRID:AB_2796601
PE Goat anti-mouse IgG Antibody (Clone Poly4053)	Biolegend	Cat# 405307; RRID:AB_315010
Mouse Anti-Human IgG1 Fc-PE (HP6001)	SouthernBiotech	Cat# 9054-09; RRID:AB_2796628
Mouse Anti-Human IgG2 Fc-PE (HP6002)	SouthernBiotech	Cat# 9070-09; RRID:AB_2796639
Goat Anti-Mouse IgG3, Human ads-AP	SouthernBiotech	Cat# 1100-09; RRID:AB_2794576
Mouse Anti-Human IgG4 Fc-PE (HP6025)	SouthernBiotech	Cat# 9200-09; RRID:AB_2796693

**Biological samples**		

Human serum from anti-A2 sensitized patients	Hôpital Erasme ULB, Brussels, Belgium	N/A
Polyclonal immunoglobulin G (from Normal Human Plasma)	Athens Research & Technology	Cat#16-16-090707

**Chemicals, peptides, and recombinant proteins**		

Protein A		
Protein G		
2-Deoxy-2-fluoro-L-fucose	Carbosynth	Cat# MD06089
HLA-A2	NIH Tetramer Facility	
HLA-A1	NIH Tetramer Facility	
HSV-gD (Ectodomain)	Immune Technology	Cat# IT-005-055p
Albumin, Bovine Fraction V (BSA)	RPI	Cat# A30075-250.0
Tween 20	Sigma Aldrich	Cat# P9416-100ML
Streptavidin Magnetic Beads	NEB	Cat# S1420S
Streptavidin-R-Phycoerythrin	Agilent	Cat# PJ31S- 1
NeutrAvidin	ThermoFisher Scientific	Cat# 31000
QUANTI-Luc	Invivogen	Cat# rep-qlc2
eBioscience™ Cell Stimulation Cocktail (500X)	ThermoFisher Scientific	Cat# 00-4970-93
LIVE/DEAD™ Fixable Violet Dead Cell Stain Kit, for 405 nm excitation	ThermoFisher Scientific	Cat# L34964
Electron Microscopy Sciences 16% Paraformaldehyde Aqueous Solution, EM Grade, Bottle 100 ML	Fisher Scientific	Cat# 15710
FcγRIIIa V158	In house (Boesch et al. 2014)^104^	N/A
FcγRI	In house (Boesch et al. 2014)^104^	N/A
Melon™ Gel IgG Spin Purification Kit	ThermoFisher Scientific	Cat# 45206
Chromabond C_18_ec beads	Macherey-Nagel	Cat#30611
Sequencing Grade Modified Trypsin	Promega	Cat#V511A
Protein G Sepharose® 4 Fast Flow beads	GE Healthcare	Cat#17-0618-05
Formic Acid	Sigma-Aldrich	Cat#94318; CAS: 64-18-6
Acetonitrile (LC-MS grade)	Biosolve	Cat#012078; CAS: 75-05-8
Trifluoroacetic Acid (LC-MS grade)	Merck	Cat#85183; CAS: 76-05-1
Ammonium Bicarbonate	Sigma-Aldrich	Cat#09830; CAS: 1066-33-7

**Critical commercial assays**		

QUANTI-Luc Lucia luciferase reporter gene system	invivogen	rep-qlc2
RNeasy kit	Qiagen	Cat# 74104

**Deposited data**		

Liquid chromatography - Mass spectrometry data	This paper	https://doi.org/10.25345/C5Q52FJ02 (Accession code: MSV000090430)

**Experimental models: Cell lines**		

Mouse HLA-A2 hybridoma cells	ATCC	HB-82 BB7.2
HEK-expi293 cells		
Jurkat Lucia NFAT-CD16 cells	Invivogen	Cat# jktl-nfat-cd16
A375 cells	ATCC	CRL-1619
NK-92 human NK cells	NantKwest/ImmunityBio	https://immunitybio.com/

**Recombinant DNA**		

pCMV-M1	Addgene	Cat# 23007

**Software and algorithms**		

Flowjo v10	Flowjo	https://www.flowjo.com/
Prism	GraphPad	https://www.graphpad.com/
Bruker Compass DataAnalysis	Bruker Daltonics	https://www.bruker.com
MSConvertGUI	ProteoWizard	https://proteowizard.sourceforge.io
LaCyTools	LaCyTools (Jansen et al. 2016)^105^	https://git.lumc.nl/cpm/lacytools
BioRender	BioRender	https://biorender.com
Microsoft Office Excel and PowerPoint	Microsoft	https://microsoft.com

**Other**		

96-well EIA/RIA clear flat bottom polystyrene high bind microplate	Corning	Cat# 3361
96 well clear bottom sterile tissue culture treated plates with lid	Thomas Scientific	Cat# 6916A05
Suspension culture microplate, 96 well, PS, U-bottom, with lid, sterile, single packed	Grenier bio-one	Cat# 650185
USA Scientific Inc 96-well PlateOne PP V-bottom plate, non-sterile	Fisher Scientific	Cat# 18339600
Octet® High Precision Streptavidin 2.0 (SAX2) Biosensors	Sartorius	Cat# 18-5136

### Resource availability

#### Lead contact

Further information and requests for resources should be directed to and will be fulfilled by the lead contact, Margaret E. Ackerman (margaret.e.ackerman@dartmouth.edu).

#### Materials availability

Unique reagents generated in this study are available from the lead contact upon request.

### Experimental model and subject details

Clinical sample collection and analysis were approved by the Ethics Review Board of the Hôpital Erasme, Brussels, Belgium. Written informed consent was obtained from study participants. Clinical and demographic characteristics were collected from the patient’s electronic medical records (Transkid-RedCap) (Tables S1 and S2). Patients with detectable anti-HLA-A2 alloantibodies, whether or not these antibodies represented a DSA, were included in the study, a criterion based on development of assays for this specificity as a model and given its clinical prevalence. By necessity, this choice excluded recipients who were HLA-A2 antigen positive as well as individuals who were seropositive for other specificities but not HLA-A2, resulting in the exclusion of many patients diagnosed with DSA and experiencing AMR. The samples tested were those collected at the time that HLA-A2 seropositivity was first diagnosed. However, variability between subjects in the time between the induction of a response and its diagnosis is expected given screening required out-of-pocket costs to patients and was therefore optional. The median interval between transplant and DSA assessment yielding a positive result for HLA-A2 was 3,657 days (interquartile range (IQR) 2,246–7,244 days). Patients without detectable anti-A2 antibodies were included as controls. Anti-HLA-A2 antibodies were detected using clinical Luminex® single antigen bead assay according to the manufacturer’s instructions (Immucor® Lifecodes). Following incubation with serum, Mean Fluorescence Intensity (MFI) of HLA antigen-coated beads was measured with a fluoroanalyser using xPonent software for data acquisition and Match It! Software (Immucor® Lifecodes) for data analysis. Positivity thresholds were defined according to the manufacturer’s instructions. For individuals with renal transplant, AMR was diagnosed based on clinical parameters (serum creatinine and proteinuria) and renal transplant biopsy, when contributive. AMR histological characteristics were glomerulitis, peritubular capillaritis, microvascular inflammation and C4d immunostaining positivity. The median interval between diagnosis of AMR and HLA-A2-specific response detection (and test sample collection) was 1,483 days (IQR 1,286–2,228 days). Samples from patients with acute rejection were taken >1 yr after treatment with plasma exchange, IVIg, and corticoids. Patients with chronic rejection received no anti-rejection treatment.

### Method details

#### Cloning and expression of recombinant HLA-A2 mAbs

Variable domain gene sequences of the heavy and the light chain (V_H_ and V_L_) of mouse HLA-A2 hybridoma cells (ATCC, USA) were defined to support recombinant production of a panel of human subclass-switched chimeric antibodies. Briefly, mRNA was isolated from hybridoma cells using the RNeasy kit (Qiagen, Germany), and cDNA generated using the VRSO cDNA kit (ThermoFisher, USA). This cDNA was then amplified using degenerate primers^106^ to selectively amplify V_H_ and V_L_ regions, which were sequenced and cross-referenced and annotated using BLAST and IMGT-based tools. Verified V_H_ and V_L_ sequences were used to design gene blocks (Twist Biosciences, USA) that contained murine V_H_, V_L_, C_L_ and C_H_1 domains paired with human hinge and C_H_2 and C_H_3 Fc domains for each IgG subtype (Table S3). These gene blocks were cloned by overlap extension into the pCMV expression plasmid.

Chimeric antibodies were transiently expressed via heavy and light chain plasmid co-transfection in HEK-expi293 cells, and purified using Protein A (IgG1, IgG2, and IgG4) or Protein G (IgG3) chromatography as previously reported.^107^^,^^108^ Afucosylated IgG1 and IgG3 were produced by adding 0.15 mM of 2-fluorofucose (2FF) substrate in the growth medium, as described.^108^

#### IgG subclass and response magnitude measurements

A custom multiplex assay was performed as previously described^109^^,^^110^ in order to define the total levels and subclass profiles of HLA-A2-, HLA-A1- (NIH Tetramer Facility, HLA-A∗02:01 complexed with either CLGGLLTMV peptide from Epstein Bar Virus membrane protein or GLCTLVAML peptide from Epstein Bar Virus mRNA export factor ICP27, HLA-A∗01:01 complexed with VTEHDTLLY from cytomegalovirus pp50), and HSV-gD- (Immune Technology, USA) specific antibodies. Briefly, antigen-coupled microspheres were diluted in Assay Buffer (PBS + 0.1% BSA + 0.05% Tween20), and mAb or serum, followed by washing and detection with R-phycoerythrin (PE)-conjugated anti-Human IgG (Southern Biotech, USA), anti-mouse IgG (Biolegend, USA) or anti-human IgG1 (Southern Biotech, USA), IgG2 (Southern Biotech, USA), IgG3 (Southern Biotech, USA) and IgG4 (Southern Biotech, USA), respectively. These detection reagents have been characterized recently for specificity and sensitivity across IgG allotypes.^111^ Median fluorescent intensities (MFI) were acquired on a FlexMap 3D (Luminex, USA).

#### Affinity purification of HLA-A2 specific antibodies

HLA-A2-specific antibodies were purified from human sera using magnetic, antigen-conjugated beads (Figure S3, Table S4). Magnetic streptavidin coated beads (NEB, S1420S) were incubated with biotinylated HLA-A2. Briefly, 50 μL streptavidin beads were washed with wash buffer (0.5 M NaCl, 20 mM Tris-HCl (pH 7.5), 1 mM EDTA) followed by incubation with 20 μg biotinylated HLA-A2 for 2 hours at room temperature or overnight at 4°C. After washing five times, beads were blocked using 200 μL heat inactivated FBS for 2 hours. For purification, 150 μL of beads were co-incubated with 50 μL of sera for 3 hours on a rotational mixer, followed by three washes using PBS-TBN (PBS-1X, 0.1% BSA, 0.02% Tween 20, 0.05% sodium azide, pH 7.4). HLA-A2-specific antibodies were eluted by resuspending beads in 50 μL of 1% formic acid (pH 2.9) and incubating on a rotational mixer for 10 min at room temperature. Supernatant was withdrawn and 20 μL of 0.5 M sodium phosphate dibasic was added to each tube to neutralize the pH. The resulting eluate was split into two parts and used for LC-MS-based IgG Fc glycosylation analysis as well as for enrichment confirmation analysis, as described above.

#### Affinity purification of total IgG antibodies

Total IgG was captured from plasma/serum using Protein G Sepharose Fast Flow 4 beads (GE Healthcare, Uppsala, Sweden) according to established procedures.^112^ Briefly, affinity beads were span down, stripped from their supernatants and resuspended in 1x phosphate-buffered saline (PBS) in a 1:24 bead slurry-to-PBS ratio. Next, 50 μL suspended beads were pipetted into each well of a 96-well filter plate (Orochem Technologies, Naperville, IL). Following three washing steps with 1x PBS, 1 μL of each plasma sample premixed in 20 μL 1x PBS were applied to the wells. Then, the plate was sealed and incubated at room temperature on a horizontal shaker at 1000 rpm for an hour, after which samples were subjected to consecutive washing steps by 1x PBS and water (thrice each). Consecutively, 100 μL of 100 mM formic acid (Sigma-Aldrich, Steinheim, Germany) solution was added into each well and the plate was incubated for 5 minutes on a horizontal shaker at 1000 rpm, which was followed by centrifuge-aided elution (100 g for 1 min) into a 96-well collection plate. Subsequently, purified total IgGs were dried by vacuum centrifugation and subjected to overnight tryptic digestion at 37°C following their resuspension in 20 μL 50 mM ammonium bicarbonate and 20 μL sequencing grade trypsin solution (25 ng per sample: Promega Corporation, WI, Madison). Following tryptic digestion, the purified total IgG glycopeptides were stored at −20°C until LC-MS analysis.

#### Preparation of HLA-A2-specific antibodies for LC-MS-based Fc glycosylation analysis

Affinity purified HLA-A2-specific IgG dried by vacuum centrifugation and subsequently redissolved in 20 water and 7 μL 1x PBS, which resulted in a pH of 8. Next, samples were subjected to tryptic digestion overnight at 37°C using sequencing grade trypsin (25 ng per sample). Then, tryptic HLA-A2 specific IgG glycopeptides were enriched and desalted by reversed-phase solid phase extraction (Chromabond C18ec beads (Marcherey-Nagel, Düren, Germany), similarly to a previous report.^96^ A 20 mg/mL supsension was obtained in 50% acetonitrile (ACN; Biosolve, Valkenswaard, Netherlands), of which 250 μL was added to the wells of a 96-well filter plate (Orochem Technologies Inc., Naperville, IL). The beads were activated by consecutive washing steps with 80% ACN 0.1% trifluoroacetic acid (TFA; Merck, Darmstadt, Netherlands), 50% ACN 0.1% TFA and finally three times 0.1% TFA (200 uL each). Then, samples were added to the filterplate in 0.1% TFA and shaken for 10 min at 500 rpm on a horizontal shaker, followed by three washing steps by 0.1% TFA (100 μL each). The bound Fc glycopeptides were firstly eluted with 100 μL of 18% ACN 0.1% TFA, and secondly with 100 uL of 50% ACN 0.1% TFA into separate 96-well V-bottom plates. Each elution step was preceded by 5 minutes incubation at 500 rpm on a horizontal shaker in the respective eluent. Subsequently, the desalted, matching HLA-A2-specific IgG glycopeptide samples from the two elution plates were pooled, and then dried by vacuum centrifugation. The dried samples were resuspended in 40 μL of 25 mM ammonium bicarbonate and stored at −20°C until LC-MS analysis.

#### LC-MS based Fc glycosylation analysis

Glycopeptides were separated with an Ultimate 3000 RSLCnano high-performance liquid chromatography system (Thermo Scientific, Waltham, MA) equipped with an Acclaim PepMap 100 trap column (100 μm × 20 mm, 5 μm particle size; Thermo Scientific) and an Acclaim PepMap RSLC C18 nano-column (75 μm × 150 mm, 2 μm particle size) analytical column. Five hundred nL of total IgG and two hundred nL of HLA-A2-specific IgG was injected and separated with a gradient from 97% solvent A (0.1% formic acid in water) and 3% solvent B (95% ACN) down to 27% solvent B, at a flowrate of 700 nL/min over 15 minutes. The LC-MS system was hyphenated to a maXis HD quadrupole time-of-flight mass spectrometer (Bruker Daltonics, Billerica, MA) via an electrospray ionization interface, which was equipped with a CaptiveSpray nanoBooster using ACN-enriched nitrogen gas (at 0.2 bar pressure and a dry gas flow rate of 3 L/min). A frequency of 1 Hz was used for recording the spectra in the m/z range of 550–1800 in positive ion polarity mode. The transfer time was set to 130 μs, the pre-pulse storage time to 10 μs, while the collision energy was set to 5 eV.^113^ This method allowed unambiguous identification of IgG Fc glycopeptides in a subclass specific manner based on accurate mass (MS1) and specific migration positions in liquid chromatography.

#### LC-MS data processing

Data processing was performed according to established procedures.^91^ Briefly, mzXML files were generated from raw LC-MS spectra using MSConvertGUI (ProteoWizard, Palo Alto, CA) and an in-house developed software, LaCyTools^105^ was used for chromatogram alignment, calibration, and targeted data extraction. The targeted glycopeptide extraction list included the 2+ and 3+ charge states and was generated by manual annotation of the mass spectra and based on literature.^114^ A commercially available polyclonal human IgG standard (isolated from normal human plasma; Athens Research & Technology, Athens, GA) and due to limited sample amount, plasma of a single HLA-A2 positive individual were prepared and measured in triplicates to assess robustness of the HLA-A2-specific method, resulting in an average intra-plate coefficient of variation (CV) of 3.7%. Overall method robustness for the total IgG method was assessed by preparing and measuring triplicate plasma samples of six HLA-A2 positive individuals, resulting in an average intra-plate CV of 1.4%. Plasma from seven HLA-A2 negative individuals were used as negative controls. All spectra below the average intensity plus once the standard deviation of negative controls was excluded from further analysis. Inclusion of an analyte for final data analysis was based on quality criteria including signal-to-noise (>9), isotopic pattern quality (<25% deviation from the theoretical isotopic pattern), and mass error (within a ±20 ppm range). Furthermore, only analytes present in at least 1 out of 4 HLA-A2-specific IgG spectra (25%) were included for relative quantification.

#### FcγR binding assay

The FcγR binding profiles of polyclonal HLA-A2-specific antibodies were defined using the Fc Array assay described previously.^76^ Briefly, HLA-A2 coated microspheres were generated as described above for the multiplex assay, serum antibodies were detected with FcγRIIIa tetramers formed by mixing biotinylated FcγRIIIa V158^104^ with a 1/4^th^ molar ratio of Streptavidin-RPE (Agilent Technologies, USA).

#### FcγRIIIa signaling assay

As a surrogate for the ADCC potential of the serum-derived and recombinant HLA-A2 specific antibodies, FcγRIIIa signaling was measured by using a Jurkat Lucia NFAT reporter cell line (InvivoGen, USA)^75^^,^^115^ in which cross-linking of antigen-bound antibodies and FcγRIIIa leads to the secretion of luciferase into the cell culture supernatant. Levels of Lucia luciferase secreted can then be directly measured by bioluminescence. Briefly, a clear flat bottom, high binding 96 well plate (Corning, USA) was coated with either 1 μg/mL NeutrAvidin (ThermoFisher Scientific, USA) or 1 μg/mL biotinylated HLA-A2 antigen via incubation at 4°C overnight. The plates were then washed with 1X phosphate-buffered Saline (PBS) plus 0.05% Tween 20 and blocked with 1x PBS plus 2.5% bovine serum albumin (BSA) for 1 hour at room temperature. HLA-A2 antigen coated plates were directly used, whereas the plates coated first with NeutrAvidin were washed and incubated with 2 μg/mL biotinylated HLA-A2 antigen for 1 hour at room temperature. After washing, 200 μL of serum diluted 1:100 and 100,000 cells/well in growth medium lacking antibiotics were added and incubated at 37°C for 24 hours. Alternatively, for HLA-A2 mAbs, the plates were first incubated with mAb for 3 hours at room temperature, washed and then was incubated with cells. The following day, 25 μL of supernatant was drawn from each well and then transferred to an opaque, white, clear bottom 96-well plate (Thomas Scientific, USA) to which 75 μL of freshly prepared QuantiLuc (InvivoGen, USA) was added. Luminescent was measured immediately on a SpectraMax Paradigm plate reader (Molecular Devices, USA) using 1 s of integration time for collecting light. The reported values are the means of three kinetic readings collected at 0, 2.5 and 5 minutes. Treatment of reporter cells with cell stimulation cocktail (ThermoFisher Scientific, USA) was used as a positive control, and an α4β7-specific mAb was used as a negative control. Baseline control wells contained assay medium instead of antibody sample.

#### Antibody dependent cellular cytotoxicity (ADCC) assay

ADCC activity of the HLA-A2 mAbs were assessed by a plate-based assay using an adaptation of a method described previously.^116^ Briefly, A375 cells which expressed GFP, obtained by transducing A375 cells (ATCC®, USA) with Ppy RE9-GFP retroviral backbone^117^ (50,000 cells/well) were added to the titrated HLA-A2 mAbs in a total of 100 μL of complete assay medium (RPMI-1640 media (Corning, USA) supplemented with 10% heat-inactivated FBS (Biowest), 1 mM sodium pyruvate (Corning, USA), 1X MEM nonessential amino acids (Corning, USA) and 1X Penicillin Streptomycin (Corning, USA)), in a sterile, U-bottom 96 well suspension culture plate (Greiner Bio-one). The plate was then incubated at 37°C at 5% CO_2_ for 30 min. NK-92 human NK cells (NantKwest, formerly Conkwest) were used as effector cells for the assay. 50 μL of NK-92 cells were then added at an effector to target ratio (E:T) of 2:1. Cells were co-cultured at 37°C at 5% CO_2_ for 3 hours. After 3 hours, contents of the wells were transferred to a 96 well V-bottom plate (USA Scientific, USA). The cells were washed with ice-cold 1X PBS following centrifugation at 400 g for 5 min at 4°C. After that, the cells were stained with live/dead fixable violet (Invitrogen, USA) for 30 minutes in ice according to the manufacturer’s instructions, and then were fixed with 4% Paraformaldehyde (Electron Microscopy Sciences, 15710) at 4°C in dark for 30 minutes, washed with PBSF (1X PBS supplemented with 0.1% BSA), and resuspended in 100 μL of PBSF. Data were acquired on a Novocyte flow cytometer and analyzed using Flowjo V10. The FSC-H and SSC-H parameters were used to gate the cells. The target population was gated as the GFP^+^ population based on SSC-H vs FITC-H biplot. The proportion of cell death was determined based on the percentage of GFP+/Violet+ cells. Data were analyzed in GraphPad Prism.

#### FcγR affinity measurements

High Precision Streptavidin 2.0 (SAX2) biosensor tips (Sartorius) were used to determine the kinetics of HLA-A2-specific IgG binding to human FcγRIIIa and FcγRI by biolayer interferometry (BLI) on an Octet (Forte Bio) instrument. Streptavidin (SA) biosensor tips were first loaded with HLA-A2 antigen, followed by HLA-A2 antibody, and finally dipped into FcγR to study the antibody-receptor kinetics. Biosensors were equilibrated with 0.05% Tween-PBS for 60 s, loaded with 0.25 μg/mL biotinylated HLA-A2 antigen (in 0.05% Tween-PBS) until they reached the threshold of 1 nm, and then dipped into 0.05% Tween-PBS to reach baseline for 60 s. After that, the biosensors were loaded with 3 μg/mL of recombinant HLA-A2 IgG (in 0.05% Tween-PBS) for 600 s, dipped into 0.05% Tween-PBS to reach baseline for 60 s, and then dipped into 5 μM monomeric FcγRIIIa for 180 s or 292 nM FcγRI for 300 s for association. To improve signal to noise, serum IgG was purified using Melon gel (Thermo) according to the manufacturer’s instructions. Polyclonal serum IgG was loaded for 2,400 s. For FcγR dissociation rate (k_d_) determination, two SAX2 biosensor tips were used, one for the measurement and one as a reference to subtract background signal changes due to dissociation of other components, such as dissociation of HLA-A2-specific IgG from biotinylated HLA. Reference biosensors loaded with IgGs were dipped into buffer (0.05% Tween-PBS) instead of receptors. Finally, the sensors were dipped into 0.05% Tween-PBS for 180 s and 300 s for the dissociation of FcγRIIIa and FcγRI, respectively. Data analysis.

### Quantification and statistical analysis

Statistical analysis was performed in GraphPad Prism version 9. Replicates and statistical tests used are reported in figure legends. A nonlinear regression using a one phase exponential decay model was used to fit the dissociation curve and calculate off-rate.

## Data Availability

• The Liquid chromatography - Mass spectrometry data generated for this study is available at https://doi.org/10.25345/C5Q52FJ02 (Accession code: MSV000090430). • Data reported in this study will be shared upon request from the lead contact. • This paper does not report original code. • Any additional information required to reanalyze the data reported in this paper is available from the lead contact upon request.

## Supplemental information

Document S1. Figures S1–S5 and Tables S1–S4

Document S2. Article plus supplemental information

## References

[1] Thongprayoon C., Hansrivijit P., Leeaphorn N., Acharya P., Torres-Ortiz A., Kaewput W., Kovvuru K., Kanduri S.R., Bathini T., Cheungpasitporn W. (2020). Recent advances and clinical outcomes of kidney transplantation. J. Clin. Med..

[2] Chong A.S., Rothstein D.M., Safa K., Riella L.V. (2019). Outstanding questions in transplantation: B cells, alloantibodies, and humoral rejection. Am. J. Transplant..

[3] Cheng E.Y., Everly M.J., Kaneku H., Banuelos N., Wozniak L.J., Venick R.S., Marcus E.A., McDiarmid S.V., Busuttil R.W., Terasaki P.I., Farmer D.G. (2017). Prevalence and clinical impact of donor-specific alloantibody among intestinal transplant recipients. Transplantation.

[4] Davis S., Cooper J.E. (2017). Acute antibody-mediated rejection in kidney transplant recipients. Transplant. Rev..

[5] Valenzuela N.M., Reed E.F. (2017). Antibody-mediated rejection across solid organ transplants: manifestations, mechanisms, and therapies. J. Clin. Invest..

[6] Black C.K., Termanini K.M., Aguirre O., Hawksworth J.S., Sosin M. (2018). Solid organ transplantation in the 21st century. Ann. Transl. Med..

[7] Gebel H.M., Kasiske B.L., Gustafson S.K., Pyke J., Shteyn E., Israni A.K., Bray R.A., Snyder J.J., Friedewald J.J., Segev D.L. (2016). Allocating deceased donor kidneys to candidates with high panel-reactive antibodies. Clin. J. Am. Soc. Nephrol..

[8] Cecka J.M. (2009). Calculated PRA (CPRA): the new measure of sensitization for transplant candidates: sensitized patients, PRA and CPRA. Am. J. Transplant..

[9] Viglietti D., Loupy A., Vernerey D., Bentlejewski C., Gosset C., Aubert O., Duong van Huyen J.P., Jouven X., Legendre C., Glotz D. (2016). Value of donor-specific anti-HLA antibody monitoring and characterization for risk stratification of kidney allograft loss. J. Am. Soc. Nephrol..

[10] Terasaki P.I., Ozawa M. (2004). Predicting kidney graft failure by HLA antibodies: a prospective trial: prediction of failure by antibodies. Am. J. Transplant..

[11] Timofeeva O.A. (2018). Donor-specific HLA antibodies as biomarkers of transplant rejection. Clin. Lab. Med..

[12] Ghandorah S., Yilmaz S., Abadi A., Rahmanian T., Wang J., Khan F.M., Berka N. (2015). Characteristics of donor-specific anti-HLA antibodies in renal transplant recipients. Hum. Immunol..

[13] Visentin J., Marroc M., Guidicelli G., Bachelet T., Nong T., Moreau J.-F., Lee J.-H., Merville P., Couzi L., Taupin J.-L. (2015). Clinical impact of preformed donor-specific denatured class I HLA antibodies after kidney transplantation. Clin. Transplant..

[14] Böhmig G.A., Eskandary F., Doberer K., Halloran P.F. (2019). The therapeutic challenge of late antibody-mediated kidney allograft rejection. Transpl. Int..

[15] Salvadori M., Bertoni E. (2013). Acute antibody-mediated rejection in kidney transplantation: clinical and therapeutic aspects. J. Nephrol. Ther..

[16] Sellarés J., Reeve J., Loupy A., Mengel M., Sis B., Skene A., de Freitas D.G., Kreepala C., Hidalgo L.G., Famulski K.S., Halloran P.F. (2013). Molecular diagnosis of antibody-mediated rejection in human kidney transplants. Am. J. Transplant..

[17] Lefaucheur C., Loupy A., Vernerey D., Duong-Van-Huyen J.P., Suberbielle C., Anglicheau D., Vérine J., Beuscart T., Nochy D., Bruneval P. (2013). Antibody-mediated vascular rejection of kidney allografts: a population-based study. Lancet.

[18] Butler C.L., Valenzuela N.M., Thomas K.A., Reed E.F. (2017). Not all antibodies are created equal: factors that influence antibody mediated rejection. J. Immunol. Res..

[19] Alelign T., Ahmed M.M., Bobosha K., Tadesse Y., Howe R., Petros B. (2018). Kidney transplantation: the challenge of human leukocyte antigen and its therapeutic strategies. J. Immunol. Res..

[20] Bray R.A., Gebel H.M., Townsend R., Roberts M.E., Polinsky M., Yang L., Meier-Kriesche H.U., Larsen C.P. (2018). De novo donor-specific antibodies in belatacept-treated vs. Cyclosporine-treated kidney transplant recipients: post-hoc analyses of the randomized phase III BENEFIT and BENEFIT-EXT studies. Am. J. Transplant..

[21] Severova G., Nikolov I., Sikole A., Cakalaroski K., Popov Z., Ivanovski N. (2018). Clinical importance of non-donor-specific HLA antibodies and possible impact on graft histology in kidney transplant recipients – 12 Months protocol biopsy study. Transplantation.

[22] Everly M.J., Rebellato L.M., Haisch C.E., Ozawa M., Parker K., Briley K.P., Catrou P.G., Bolin P., Kendrick W.T., Kendrick S.A. (2013). Incidence and impact of de novo donor-specific alloantibody in primary renal allografts. Transplantation.

[23] Hart A., Smith J.M., Skeans M.A., Gustafson S.K., Wilk A.R., Castro S., Foutz J., Wainright J.L., Snyder J.J., Kasiske B.L., Israni A.K. (2020). OPTN/SRTR 2018 annual data report: kidney. Am. J. Transplant..

[24] Kwun J., Knechtle S. (2020). Experimental modeling of desensitization: what have we learned about preventing AMR?. Am. J. Transplant..

[25] Chandra A., Midtvedt K., Åsberg A., Eide I.A. (2019). Immunosuppression and reproductive health after kidney transplantation. Transplantation.

[26] Kramer C.S.M., Franke-van Dijk M.E.I., Priddey A.J., Pongrácz T., Gnudi E., Car H., Karahan G.E., van Beelen E., Zilvold-van den Oever C.C.C., Rademaker H.J. (2019). Recombinant human monoclonal HLA antibodies of different IgG subclasses recognising the same epitope: excellent tools to study differential effects of donor-specific antibodies. Hla.

[27] Lefaucheur C., Viglietti D., Bentlejewski C., Duong van Huyen J.P., Vernerey D., Aubert O., Verine J., Jouven X., Legendre C., Glotz D. (2016). IgG donor-specific anti-human HLA antibody subclasses and kidney allograft antibody-mediated injury. J. Am. Soc. Nephrol..

[28] Valenzuela N.M., Hickey M.J., Reed E.F. (2016). Antibody subclass repertoire and graft outcome following solid organ transplantation. Front. Immunol..

[29] O'Leary J.G., Kaneku H., Banuelos N., Jennings L.W., Klintmalm G.B., Terasaki P.I. (2015). Impact of IgG3 subclass and C1q-fixing donor-specific HLA alloantibodies on rejection and survival in liver transplantation. Am. J. Transplant..

[30] Everly M.J., Rebellato L.M., Haisch C.E., Briley K.P., Bolin P., Kendrick W.T., Kendrick S.A., Morgan C., Maldonado A.Q., Harland R.C., Terasaki P.I. (2014). Impact of IgM and IgG3 anti-HLA alloantibodies in primary renal allograft recipients. Transplantation.

[31] Freitas M.C.S., Rebellato L.M., Ozawa M., Nguyen A., Sasaki N., Everly M., Briley K.P., Haisch C.E., Bolin P., Parker K. (2013). The role of immunoglobulin-G subclasses and C1q in de novo HLA-DQ donor-specific antibody kidney transplantation outcomes. Transplantation.

[32] van Osch T.L.J., Nouta J., Derksen N.I.L., van Mierlo G., van der Schoot C.E., Wuhrer M., Rispens T., Vidarsson G. (2021). Fc galactosylation promotes hexamerization of human IgG1, leading to enhanced classical complement activation. J. Immunol..

[33] Niwa R., Natsume A., Uehara A., Wakitani M., Iida S., Uchida K., Satoh M., Shitara K. (2005). IgG subclass-independent improvement of antibody-dependent cellular cytotoxicity by fucose removal from Asn297-linked oligosaccharides. J. Immunol. Methods.

[34] Ferrara C., Brünker P., Suter T., Moser S., Püntener U., Umaña P. (2006). Modulation of therapeutic antibody effector functions by glycosylation engineering: influence of Golgi enzyme localization domain and co-expression of heterologous beta1, 4-N-acetylglucosaminyltransferase III and Golgi alpha-mannosidase II. Biotechnol. Bioeng..

[35] Dekkers G., Treffers L., Plomp R., Bentlage A.E.H., de Boer M., Koeleman C.A.M., Lissenberg-Thunnissen S.N., Visser R., Brouwer M., Mok J.Y. (2017). Decoding the human immunoglobulin G-glycan repertoire reveals a spectrum of Fc-receptor- and complement-mediated-effector activities. Front. Immunol..

[36] Lofano G., Gorman M.J., Yousif A.S., Yu W.-H., Fox J.M., Dugast A.-S., Ackerman M.E., Suscovich T.J., Weiner J., Barouch D. (2018). Antigen-specific antibody Fc glycosylation enhances humoral immunity via the recruitment of complement. Sci. Immunol..

[37] Kaneko Y., Nimmerjahn F., Ravetch J.V. (2006). Anti-inflammatory activity of immunoglobulin G resulting from Fc sialylation. Science.

[38] Gunn B.M., Roy V., Karim M.M., Hartnett J.N., Suscovich T.J., Goba A., Momoh M., Sandi J.D., Kanneh L., Andersen K.G. (2019). Survivors of Ebola virus disease develop polyfunctional antibody responses. J. Infect. Dis..

[39] Lu L.L., Suscovich T.J., Fortune S.M., Alter G. (2018). Beyond binding: antibody effector functions in infectious diseases. Nat. Rev. Immunol..

[40] Plomp R., Ruhaak L.R., Uh H.-W., Reiding K.R., Selman M., Houwing-Duistermaat J.J., Slagboom P.E., Beekman M., Wuhrer M. (2017). Subclass-specific IgG glycosylation is associated with markers of inflammation and metabolic health. Sci. Rep..

[41] Wang T.T., Sewatanon J., Memoli M.J., Wrammert J., Bournazos S., Bhaumik S.K., Pinsky B.A., Chokephaibulkit K., Onlamoon N., Pattanapanyasat K. (2017). IgG antibodies to dengue enhanced for FcγRIIIA binding determine disease severity. Science.

[42] Moore J.S., Wu X., Kulhavy R., Tomana M., Novak J., Moldoveanu Z., Brown R., Goepfert P.A., Mestecky J. (2005). Increased levels of galactose-deficient IgG in sera of HIV-1-infected individuals. Aids.

[43] Raux M., Finkielsztejn L., Salmon-Cron D., Bouchez H., Excler J.L., Dulioust E., Grouin J.M., Sicard D., Blondeau C. (2000). IgG subclass distribution in serum and various mucosal fluids of HIV type 1-infected subjects. AIDS Res. Hum. Retroviruses.

[44] Kapur R., Kustiawan I., Vestrheim A., Koeleman C.A.M., Visser R., Einarsdottir H.K., Porcelijn L., Jackson D., Kumpel B., Deelder A.M. (2014). A prominent lack of IgG1-Fc fucosylation of platelet alloantibodies in pregnancy. Blood.

[45] Bakchoul T., Greinacher A., Sachs U.J., Krautwurst A., Renz H., Harb H., Bein G., Newman P.J., Santoso S. (2013). Inhibition of HPA-1a alloantibody-mediated platelet destruction by a deglycosylated anti–HPA-1a monoclonal antibody in mice: toward targeted treatment of fetal-alloimmune thrombocytopenia. Blood.

[46] Sellarés J., de Freitas D.G., Mengel M., Reeve J., Einecke G., Sis B., Hidalgo L.G., Famulski K., Matas A., Halloran P.F. (2011). Understanding the causes of kidney transplant failure: the dominant role of antibody-mediated rejection and nonadherence. Am. J. Transplant..

[47] Lefaucheur C., Loupy A., Hill G.S., Andrade J., Nochy D., Antoine C., Gautreau C., Charron D., Glotz D., Suberbielle-Boissel C. (2010). Preexisting donor-specific HLA antibodies predict outcome in kidney transplantation. J. Am. Soc. Nephrol..

[48] Loupy A., Suberbielle-Boissel C., Hill G.S., Lefaucheur C., Anglicheau D., Zuber J., Martinez F., Thervet E., Méjean A., Charron D. (2009). Outcome of subclinical antibody-mediated rejection in kidney transplant recipients with preformed donor-specific antibodies. Am. J. Transplant..

[49] Khovanova N., Daga S., Shaikhina T., Krishnan N., Jones J., Zehnder D., Mitchell D., Higgins R., Briggs D., Lowe D. (2015). Subclass analysis of donor HLA-specific IgG in antibody-incompatible renal transplantation reveals a significant association of IgG4 with rejection and graft failure. Transpl. Int..

[50] Grafals M., Thurman J.M. (2019). The role of complement in organ transplantation. Front. Immunol..

[51] Navas A., Molina J., Agüera M.L., Guler I., Jurado A., Rodríguez-Benot A., Alonso C., Solana R. (2019). Characterization of the C1q-binding ability and the IgG1-4 subclass profile of preformed anti-HLA antibodies by solid-phase assays. Front. Immunol..

[52] Thurman J.M., Panzer S.E., Le Quintrec M. (2019). The role of complement in antibody mediated transplant rejection. Mol. Immunol..

[53] Biglarnia A.-R., Huber-Lang M., Mohlin C., Ekdahl K.N., Nilsson B. (2018). The multifaceted role of complement in kidney transplantation. Nat. Rev. Nephrol..

[54] Lee H., Han E., Choi A.-R., Ban T.H., Chung B.H., Yang C.W., Choi Y.J., Oh E.-J. (2018). Clinical impact of complement (C1q, C3d) binding de novo donor-specific HLA antibody in kidney transplant recipients. PLoS One.

[55] Loupy A., Lefaucheur C., Vernerey D., Prugger C., Duong van Huyen J.P., Mooney N., Suberbielle C., Frémeaux-Bacchi V., Méjean A., Desgrandchamps F. (2013). Complement-binding anti-HLA antibodies and kidney-allograft survival. N. Engl. J. Med..

[56] Guidicelli G., Guerville F., Lepreux S., Wiebe C., Thaunat O., Dubois V., Visentin J., Bachelet T., Morelon E., Nickerson P. (2016). Non-complement-binding de novo donor-specific anti-HLA antibodies and kidney allograft survival. J. Am. Soc. Nephrol..

[57] Malheiro J., Tafulo S., Dias L., Martins L.S., Fonseca I., Beirão I., Castro-Henriques A., Cabrita A. (2017). Determining donor-specific antibody C1q-binding ability improves the prediction of antibody-mediated rejection in human leucocyte antigen-incompatible kidney transplantation. Transpl. Int..

[58] Sablik K.A., Litjens N.H.R., Klepper M., Betjes M.G.H. (2019). Increased CD16 expression on NK cells is indicative of antibody-dependent cell-mediated cytotoxicity in chronic-active antibody-mediated rejection. Transpl. Immunol..

[59] Parkes M.D., Halloran P.F., Hidalgo L.G. (2017). Evidence for CD16a-mediated NK cell stimulation in antibody-mediated kidney transplant rejection. Transplantation.

[60] Yazdani S., Callemeyn J., Gazut S., Lerut E., de Loor H., Wevers M., Heylen L., Saison C., Koenig A., Thaunat O. (2019). Natural killer cell infiltration is discriminative for antibody-mediated rejection and predicts outcome after kidney transplantation. Kidney Int..

[61] Lamarthée B., Callemeyn J., Herck Y.V., Antoranz A., Anglicheau D., Becker J.U., Debyser T., Smet F.D., Vusser K.D., Eloudzeri M. (2022). Transcriptional and spatial profiling of the kidney allograft unravels a central role for FcyRIII+ innate immune cells in rejection. Preprint at medRxiv.

[62] Wiebe C., Gibson I.W., Blydt-Hansen T.D., Pochinco D., Birk P.E., Ho J., Karpinski M., Goldberg A., Storsley L., Rush D.N., Nickerson P.W. (2015). Rates and determinants of progression to graft failure in kidney allograft recipients with de novo donor-specific antibody: post- dn DSA clinical histologic progression. Am. J. Transplant..

[63] Wiebe C., Gibson I.W., Blydt-Hansen T.D., Karpinski M., Ho J., Storsley L.J., Goldberg A., Birk P.E., Rush D.N., Nickerson P.W. (2012). Evolution and clinical pathologic correlations of de novo donor-specific HLA antibody post kidney transplant: clinical pathologic correlations of de novo DSA. Am. J. Transplant..

[64] Yamashita M., Haas M. (2019). Transplant glomerulopathy with glomerular C3 deposits: why the worse outcome?. Kidney Int. Rep..

[65] Molina J., Navas A., Agüera M.L., Rodelo-Haad C., Alonso C., Rodríguez-Benot A., Aljama P., Solana R. (2017). Impact of preformed donor-specific anti-human leukocyte antigen antibody C1q-binding ability on kidney allograft outcome. Front. Immunol..

[66] Gupta G., Abu Jawdeh B.G., Racusen L.C., Bhasin B., Arend L.J., Trollinger B., Kraus E., Rabb H., Zachary A.A., Montgomery R.A., Alachkar N. (2014). Late antibody-mediated rejection in renal allografts: outcome after conventional and novel therapies. Transplantation.

[67] Piazza A., Poggi E., Ozzella G., Adorno D. (2013). Post-transplant development of C1q-positive HLA antibodies and kidney graft survival. Clin. Transpl..

[68] Wang T.T., Ravetch J.V. (2019). Functional diversification of IgGs through Fc glycosylation. J. Clin. Invest..

[69] Thomann M., Reckermann K., Reusch D., Prasser J., Tejada M.L. (2016). Fc-galactosylation modulates antibody-dependent cellular cytotoxicity of therapeutic antibodies. Mol. Immunol..

[70] Abès R., Teillaud J.-L. (2010). Impact of glycosylation on effector functions of therapeutic IgG. Pharmaceuticals.

[71] Raju T.S. (2008). Terminal sugars of Fc glycans influence antibody effector functions of IgGs. Curr. Opin. Immunol..

[72] Shinkawa T., Nakamura K., Yamane N., Shoji-Hosaka E., Kanda Y., Sakurada M., Uchida K., Anazawa H., Satoh M., Yamasaki M. (2002). The absence of fucose but not the presence of galactose or bisecting N -acetylglucosamine of human IgG1 complex-type oligosaccharides shows the critical role of enhancing antibody-dependent cellular cytotoxicity. J. Biol. Chem..

[73] Elkhalifa M.Y., Alhababi D., Saleh S.A., Khan A., Ashur Z., Alshaibi N. (2017). P221 “Naturally occuring” anti-HLA antibodies. Hum. Immunol..

[74] Morales-Buenrostro L.E., Terasaki P.I., Marino-Vázquez L.A., Lee J.H., El-Awar N., Alberú J. (2008). “Natural” human leukocyte antigen antibodies found in nonalloimmunized healthy males. Transplantation.

[75] Butler S.E., Crowley A.R., Natarajan H., Xu S., Weiner J.A., Bobak C.A., Mattox D.E., Lee J., Wieland-Alter W., Connor R.I. (2021). Distinct features and functions of systemic and mucosal humoral immunity among SARS-CoV-2 convalescent individuals. Front. Immunol..

[76] Brown E.P., Dowell K.G., Boesch A.W., Normandin E., Mahan A.E., Chu T., Barouch D.H., Bailey-Kellogg C., Alter G., Ackerman M.E. (2017). Multiplexed Fc array for evaluation of antigen-specific antibody effector profiles. J. Immunol. Methods.

[77] Beyar R. (2011). Challenges in organ transplantation. Rambam Maimonides Med. J..

[78] Laupacis A., Keown P., Pus N., Krueger H., Ferguson B., Wong C., Muirhead N. (1996). A study of the quality of life and cost-utility of renal transplantation. Kidney Int..

[79] Sethi S., Choi J., Toyoda M., Vo A., Peng A., Jordan S.C. (2017). Desensitization: overcoming the immunologic barriers to transplantation. J. Immunol. Res..

[80] Tait B.D. (2016). Detection of HLA antibodies in organ transplant recipients – triumphs and challenges of the solid phase bead assay. Front. Immunol..

[81] Zachary A.A., Leffell M.S. (2008). Detecting and monitoring human leukocyte antigen–specific antibodies. Hum. Immunol..

[82] Malard-Castagnet S., Dugast E., Degauque N., Pallier A., Soulillou J.P., Cesbron A., Giral M., Harb J., Brouard S. (2016). Sialylation of antibodies in kidney recipients with de novo donor specific antibody, with or without antibody mediated rejection. Hum. Immunol..

[83] Arnold M.L., Ntokou I.S., Doxiadis I.I.N., Spriewald B.M., Boletis J.N., Iniotaki A.G. (2014). Donor-specific HLA antibodies: evaluating the risk for graft loss in renal transplant recipients with isotype switch from complement fixing IgG1/IgG3 to noncomplement fixing IgG2/IgG4 anti-HLA alloantibodies. Transpl. Int..

[84] Jennewein M.F., Alter G. (2017). The immunoregulatory roles of antibody glycosylation. Trends Immunol..

[85] Li T., DiLillo D.J., Bournazos S., Giddens J.P., Ravetch J.V., Wang L.X. (2017). Modulating IgG effector function by Fc glycan engineering. Proc. Natl. Acad. Sci. USA.

[86] Vidarsson G., Dekkers G., Rispens T. (2014). IgG subclasses and allotypes: from structure to effector functions. Front. Immunol..

[87] Ackerman M.E., Crispin M., Yu X., Baruah K., Boesch A.W., Harvey D.J., Dugast A.S., Heizen E.L., Ercan A., Choi I. (2013). Natural variation in Fc glycosylation of HIV-specific antibodies impacts antiviral activity. J. Clin. Invest..

[88] Bournazos S., Vo H.T.M., Duong V., Auerswald H., Ly S., Sakuntabhai A., Dussart P., Cantaert T., Ravetch J.V. (2021). Antibody fucosylation predicts disease severity in secondary dengue infection. Science.

[89] Larsen M.D., de Graaf E.L., Sonneveld M.E., Plomp H.R., Nouta J., Hoepel W., Chen H.J., Linty F., Visser R., Brinkhaus M. (2021). Afucosylated IgG characterizes enveloped viral responses and correlates with COVID-19 severity. Science.

[90] Gudelj I., Lauc G., Pezer M. (2018). Immunoglobulin G glycosylation in aging and diseases. Cell. Immunol..

[91] Pongracz T., Nouta J., Wang W., van Meijgaarden K.E., Linty F., Vidarsson G., Joosten S.A., Ottenhoff T.H.M., Hokke C.H., de Vries J.J.C. (2022). Immunoglobulin G1 Fc glycosylation as an early hallmark of severe COVID-19. EBioMedicine.

[92] Chakraborty S., Gonzalez J., Edwards K., Mallajosyula V., Buzzanco A.S., Sherwood R., Buffone C., Kathale N., Providenza S., Xie M.M. (2021). Proinflammatory IgG Fc structures in patients with severe COVID-19. Nat. Immunol..

[93] Chakraborty S., Gonzalez J.C., Sievers B.L., Mallajosyula V., Chakraborty S., Dubey M., Ashraf U., Cheng B.Y.-L., Kathale N., Tran K.Q.T. (2022). Early non-neutralizing, afucosylated antibody responses are associated with COVID-19 severity. Sci. Transl. Med..

[94] Lamarthée B., Callemeyn J., Herck Y.V., Antoranz A., Anglicheau D., Becker J.U., Debyser T., Smet F.D., Vusser K.D., Eloudzeri M. (2022). Transcriptional and spatial profiling of the kidney allograft unravels a central role for FcyRIII+ innate immune cells in rejection. Preprint at medRxiv.

[95] Hoepel W., Chen H.J., Geyer C.E., Allahverdiyeva S., Manz X.D., de Taeye S.W., Aman J., Mes L., Steenhuis M., Griffith G.R. (2021). High titers and low fucosylation of early human anti-SARS-CoV-2 IgG promote inflammation by alveolar macrophages. Sci. Transl. Med..

[96] Kapur R., Della Valle L., Sonneveld M., Hipgrave Ederveen A., Visser R., Ligthart P., de Haas M., Wuhrer M., van der Schoot C.E., Vidarsson G. (2014). Low anti-RhD IgG-Fc-fucosylation in pregnancy: a new variable predicting severity in haemolytic disease of the fetus and newborn. Br. J. Haematol..

[97] Wang T.T., Sewatanon J., Memoli M.J., Wrammert J., Bournazos S., Bhaumik S.K., Pinsky B.A., Chokephaibulkit K., Onlamoon N., Pattanapanyasat K. (2017). IgG antibodies to dengue enhanced for FcgammaRIIIA binding determine disease severity. Science.

[98] Wei B., Gao X., Cadang L., Izadi S., Liu P., Zhang H.M., Hecht E., Shim J., Magill G., Pabon J.R. (2021). Fc galactosylation follows consecutive reaction kinetics and enhances immunoglobulin G hexamerization for complement activation. mAbs.

[99] Ackerman M.E., Barouch D.H., Alter G. (2017). Systems serology for evaluation of HIV vaccine trials. Immunol. Rev..

[100] Alter G., Dowell K.G., Brown E.P., Suscovich T.J., Mikhailova A., Mahan A.E., Walker B.D., Nimmerjahn F., Bailey-Kellogg C., Ackerman M.E. (2018). High-resolution definition of humoral immune response correlates of effective immunity against HIV. Mol. Syst. Biol..

[101] Loos C., Lauffenburger D.A., Alter G. (2020). Dissecting the antibody-OME: past, present, and future. Curr. Opin. Immunol..

[102] Wang T.T., Maamary J., Tan G.S., Bournazos S., Davis C.W., Krammer F., Schlesinger S.J., Palese P., Ahmed R., Ravetch J.V. (2015). Anti-HA glycoforms drive B cell affinity selection and determine influenza vaccine efficacy. Cell.

[103] Petrović T., Vijay A., Vučković F., Trbojević-Akmačić I., Ollivere B.J., Marjanović D., Bego T., Prnjavorac B., Đerek L., Markotić A. (2022). IgG N-glycome changes during the course of severe COVID-19: an observational study. EBioMedicine.

[104] Boesch A.W., Brown E.P., Cheng H.D., Ofori M.O., Normandin E., Nigrovic P.A., Alter G., Ackerman M.E. (2014). Highly parallel characterization of IgG Fc binding interactions. mAbs.

[105] Jansen B.C., Falck D., de Haan N., Hipgrave Ederveen A.L., Razdorov G., Lauc G., Wuhrer M. (2016). LaCyTools: a targeted liquid chromatography–mass spectrometry data processing package for relative quantitation of glycopeptides. J. Proteome Res..

[106] Babrak L., McGarvey J.A., Stanker L.H., Hnasko R. (2017). Identification and verification of hybridoma-derived monoclonal antibody variable region sequences using recombinant DNA technology and mass spectrometry. Mol. Immunol..

[107] de Taeye S.W., Bentlage A.E.H., Mebius M.M., Meesters J.I., Lissenberg-Thunnissen S., Falck D., Sénard T., Salehi N., Wuhrer M., Schuurman J. (2020). FcγR binding and ADCC activity of human IgG allotypes. Front. Immunol..

[108] Dekkers G., Plomp R., Koeleman C.A.M., Visser R., von Horsten H.H., Sandig V., Rispens T., Wuhrer M., Vidarsson G. (2016). Multi-level glyco-engineering techniques to generate IgG with defined Fc-glycans. Sci. Rep..

[109] Brown E.P., Normandin E., Osei-Owusu N.Y., Mahan A.E., Chan Y.N., Lai J.I., Vaccari M., Rao M., Franchini G., Alter G., Ackerman M.E. (2015). Microscale purification of antigen-specific antibodies. J. Immunol. Methods.

[110] Brown E.P., Licht A.F., Dugast A.-S., Choi I., Bailey-Kellogg C., Alter G., Ackerman M.E. (2012). High-throughput, multiplexed IgG subclassing of antigen-specific antibodies from clinical samples. J. Immunol. Methods.

[111] Crowley A.R., Richardson S.I., Tuyishime M., Jennewein M., Bailey M.J., Lee J., Alter G., Ferrari G., Morris L., Ackerman M.E. (2022). Functional consequences of allotypic polymorphisms in human immunoglobulin G subclasses. Immunogenetics.

[112] Falck D., Jansen B.C., de Haan N., Wuhrer M. (2017). High-throughput analysis of IgG Fc glycopeptides by LC-MS. Methods Mol. Biol..

[113] de Haan N., Reiding K.R., Krištić J., Hipgrave Ederveen A.L., Lauc G., Wuhrer M. (2017). The N-glycosylation of mouse immunoglobulin G (IgG)-Fragment crystallizable differs between IgG subclasses and strains. Front. Immunol..

[114] Clerc F., Reiding K.R., Jansen B.C., Kammeijer G.S.M., Bondt A., Wuhrer M. (2016). Human plasma protein N-glycosylation. Glycoconj. J..

[115] Thulin N.K., Brewer R.C., Sherwood R., Bournazos S., Edwards K.G., Ramadoss N.S., Taubenberger J.K., Memoli M., Gentles A.J., Jagannathan P. (2020). Maternal anti-dengue IgG fucosylation predicts susceptibility to dengue disease in infants. Cell Rep..

[116] González-González E., Camacho-Sandoval R., Jiménez-Uribe A., Montes-Luna A., Cortés-Paniagua I., Sánchez-Morales J., Muñoz-García L., Tenorio-Calvo A.V., López-Morales C.A., Velasco-Velázquez M.A. (2019). Validation of an ADCC assay using human primary natural killer cells to evaluate biotherapeutic products bearing an Fc region. J. Immunol. Methods.

[117] Zhang T., Wu M.-R., Sentman C.L. (2012). An NKp30-based chimeric antigen receptor promotes T cell effector functions and antitumor efficacy in vivo. J. Immunol..

