# Supplementary material for: Afucosylation of HLA-specific IgG1 as a potential predictor of antibody pathogenicity in kidney transplantation
Source: Cell Rep Med. 2022 Nov 15;3(11):100818. doi: 10.1016/j.xcrm.2022.100818 (PMC9729883; doi:10.1016/j.xcrm.2022.100818)
Supplement: Document S1. Figures S1–S5 and Tables S1–S4 [file mmc1.pdf]

Cell Reports Medicine, Volume *3*

## Supplemental information

# Afucosylation of HLA-specific IgG1

# as a potential predictor of antibody pathogenicity

# in kidney transplantation

**Pranay Bharadwaj, Sweta Shrestha, Tamas Pongracz, Catalano Concetta, Shilpee Sharma, Alain Le Moine, Noortje de Haan, Naoka Murakami, Leonardo V. Riella, Vanda Holovska, Manfred Wuhrer, Arnaud Marchant, and Margaret E. Ackerman**

| **Supplemental Figures** | |
|---|---|
| Supplemental Figure 1 | mAb controls for subclassing assay, Related to Figure 1 |
| Supplemental Figure 2 | Relationship between HLA-A2 specific IgG signal and signal reported from the clinical testing, Related to Figure 1 |
| Supplemental Figure 3 | Antigen specific antibody purification, Related to Figure 2 and STAR Methods |
| Supplemental Figure 4 | Antibody-Receptor dissociation kinetics by Biolayer Interferometry (BLI), Related to Figure 3 |
| Supplemental Figure 5 | HLA-A2 specific antibodies characteristics in patient serum samples, Related to Figures 4 and 5. |
| **Supplemental Tables** | |
| Supplemental Table 1 | Whole cohort characteristics, Related to Figure 1 |
| Supplemental Table 2 | DSA cohort characteristics, Related to Figures 4 and 5 |
| Supplemental Table 3 | HLA-A2-specific monoclonal antibody gene sequences, Related to Figures 1 and 3 |
| Supplemental Table 4 | Details of HLA molecules used for the purification of HLA-specific IgGs, Related to Figure 2 |

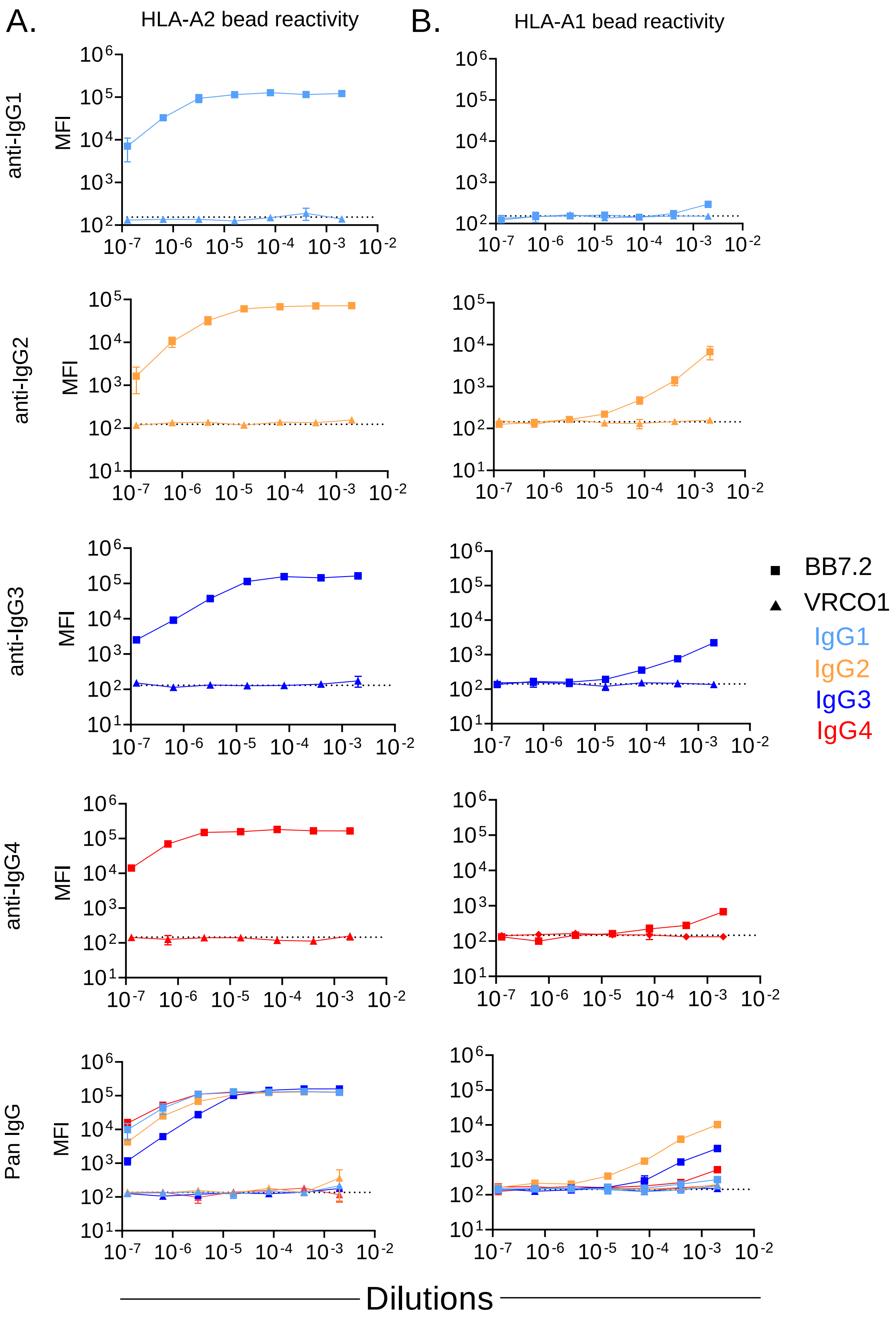


**Supplemental Figure 1. mAb controls for subclassing assay, Related to Figure 1. A-B.** Dose response profiles of HLA-A2 (positive control) and VRCO1 (negative control) mAbs to HLA-A2 (**A**) and HLA-A1 (**B**) antigens across IgG subclasses. HLA-A2-specific BB7.2 (square) and HIV-specific VRCO1 (triangle) mAb subclass controls are shown for each subclass; IgG1 (light blue), IgG2 (orange), IgG3 (dark blue) and IgG4 (red). Baseline (buffer only control) signal is indicated by dotted lines.

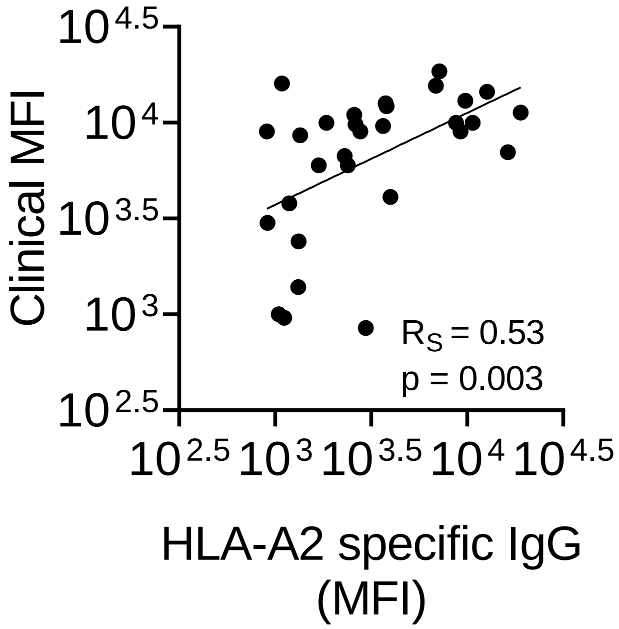


**Supplemental Figure 2. Relationship between HLA-A2 specific IgG signal and signal reported from the clinical testing, Related to Figure 1.** Correlation between HLA-A2 specific IgG MFI and values reported from the clinical testing (n=30). Spearman rank correlation ($R_S$) and corresponding *p*-value are shown in inset.

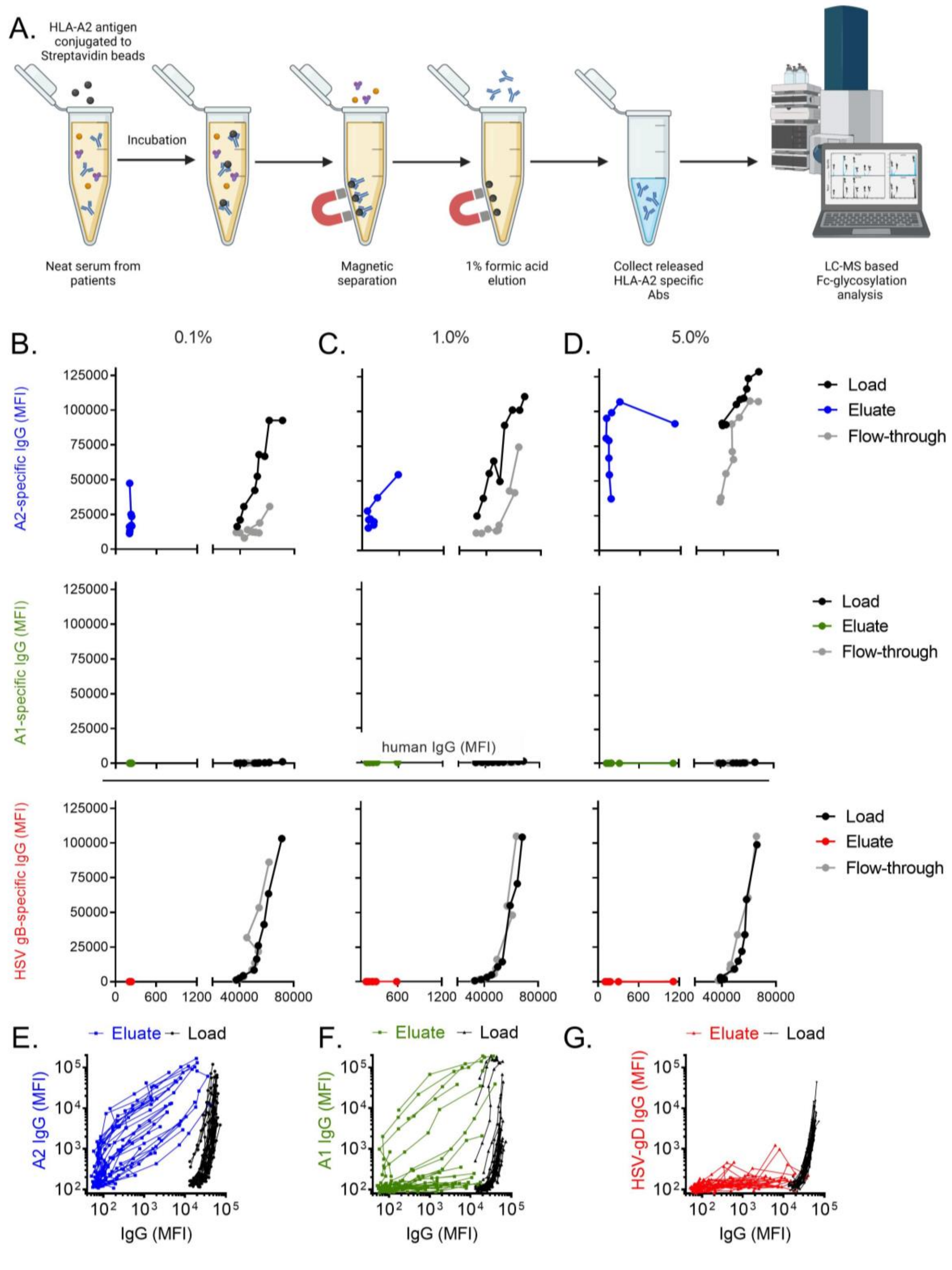


**Supplemental Figure 3. Antigen specific antibody purification, Related to Figure 2 and STAR Methods. A.** Schematic of affinity purification and Fc-glycosylation analysis of HLA-A2 specific antibodies. HLA-A2 specific antibodies were purified using HLA-A2 antigen coated magnetic beads. Diluted serum was incubated with magnetic beads to allow binding of HLA-A2-specific antibodies. Beads were washed and then the bound antibodies were eluted. Fc-glycosylation analysis of the eluted HLA-A2 specific antibodies was performed by liquid chromatography – mass spectrometry on the glycopeptide level following tryptic digestion. This figure was created with http://BioRender.com. **B-D**. Proof of concept for antigen specific antibody purification**.** Reactivity of IVIG spiked with 0.1% (**B**), 1.0% (**C**) and 5.0% (**D**) murine HLA-A2 mAb to HLA-A2 (top), HLA-A1 (middle) and HSV-gD (bottom) antigens. Load and Flow-through are shown in black and grey, respectively. Reactivity of Eluate for HLA-A2, HLA-A1 and HSV-gD are shown in blue, green, and red, respectively. **E-G.** Antigen specific antibody purification profiles. Reactivity of purified HLA-A2 specific antibodies to HLA-A2 (**E**), HLA-A1 (**F**) and HSV-gD (**G**) antigens from HLA-A2 positive (n=29) individuals. Signal for each antigen specificity in plotted on the y-axis and signal from total IgG on the x-axis. Profiles of the serum antibodies before purification (loads) are shown in black.

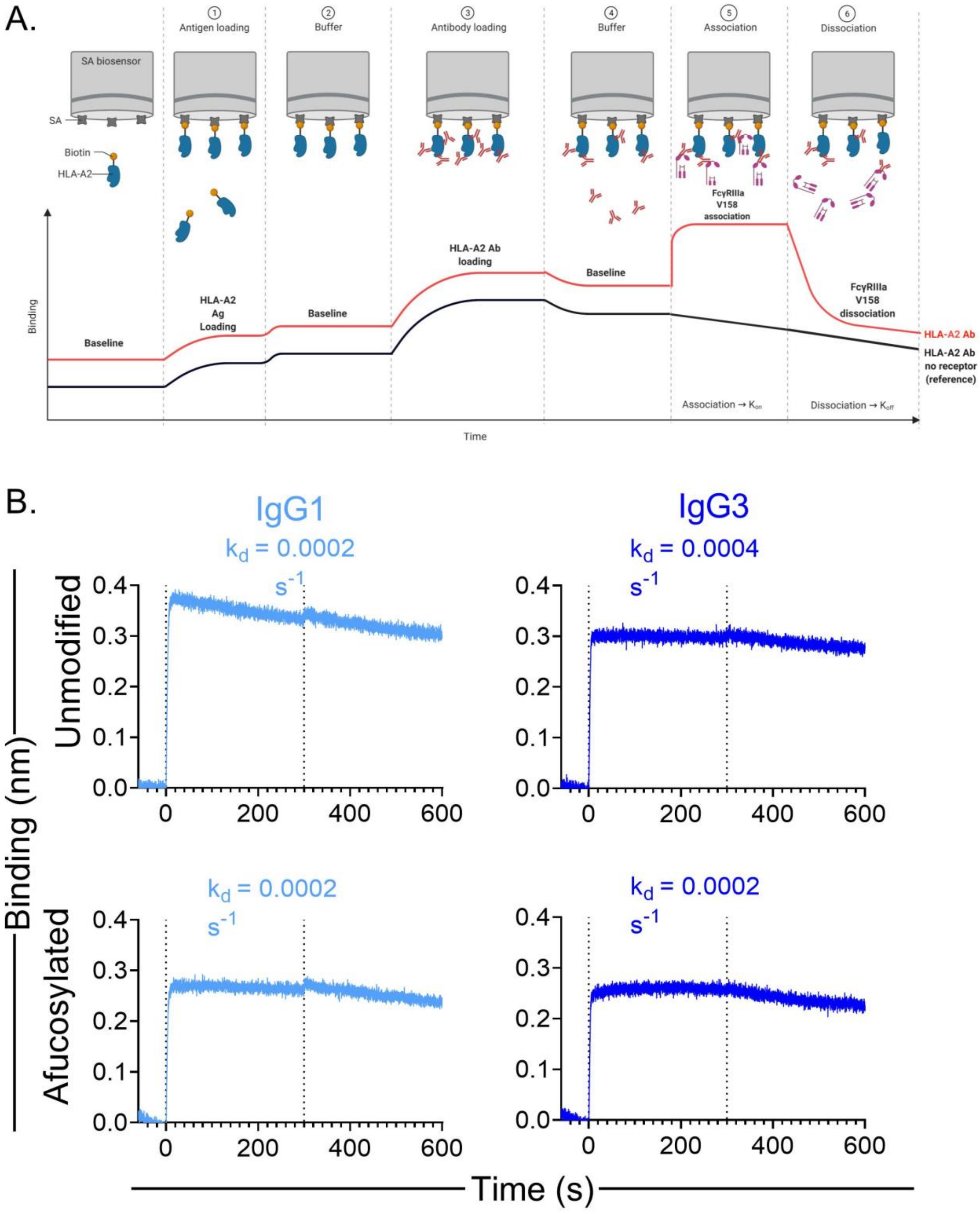


**Supplemental Figure 4. Antibody-Receptor dissociation kinetics measurement by Biolayer Interferometry (BLI), Related to Figure 3. A.** Schematic of the Antibody-Receptor dissociation kinetics measurement by Biolayer Interferometry (BLI). Streptavidin tips were first coated with biotinylated HLA-A2 antigens. After establishing a signal baseline, the tips were loaded with HLA-A2-specific antibodies, dipped into the buffer for baseline, and then finally dipped into a solution of recombinantly expressed FcγRIIIa V158 monomers. Following receptor association, tips were dipped into a buffer, which allowed receptor dissociation. The dissociation rate of FcγR was defined relatively to a reference tip which was not dipped into the receptor. This figure was created with http://BioRender.com. **B.** FcγRI binding characterization of unmodified and afucosylated HLA-A2 IgG1 and IgG3 mAbs. FcγRI association with and dissociation from unmodified and afucosylated HLA-A2 mAbs. Dissociation rates ($k_d$) are shown in inset.

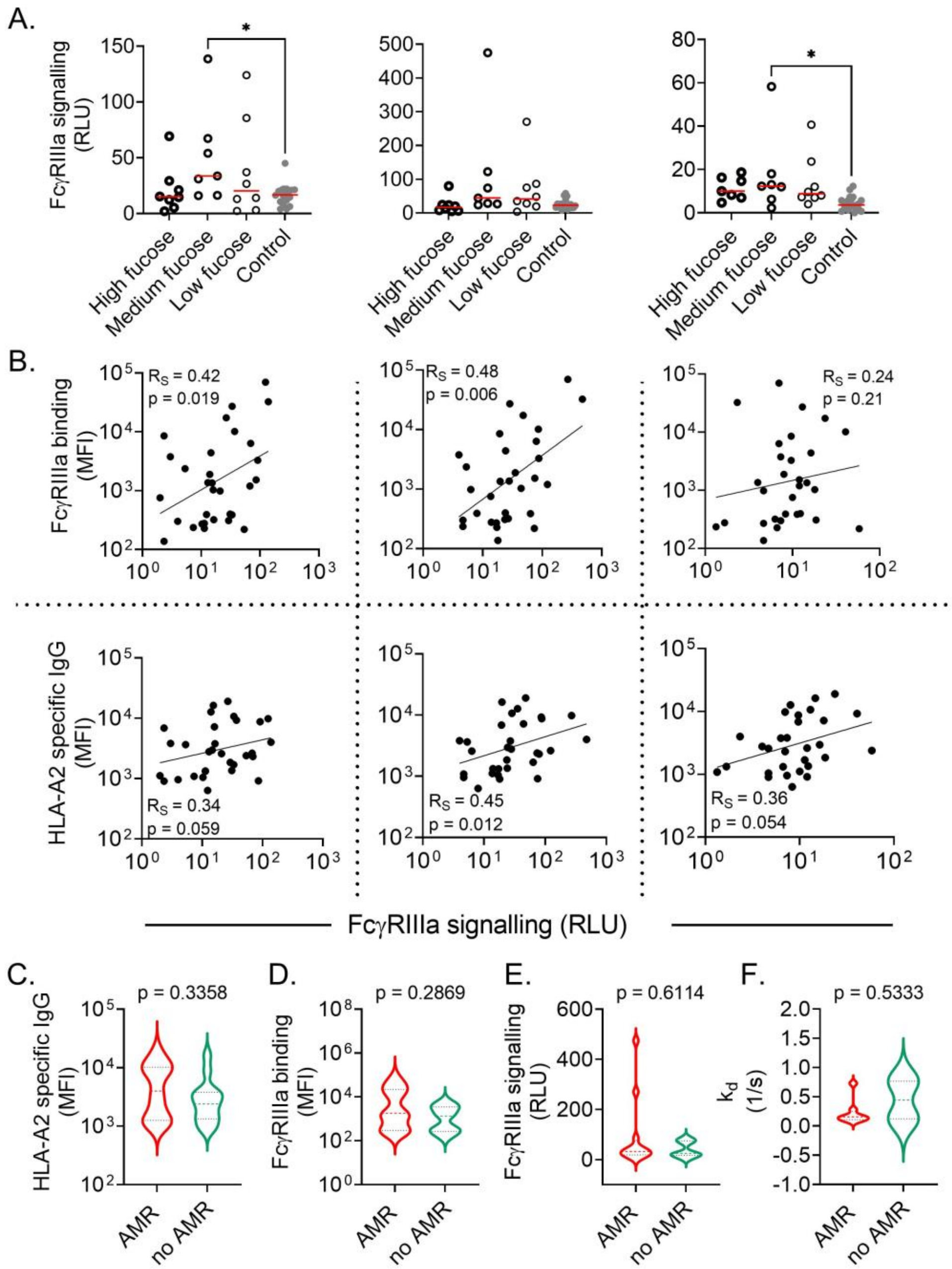


**Supplemental Figure 5. HLA-A2 specific antibodies characteristics in patient serum samples, Related to Figure 4 and 5. A-B**. FcγRIIIA V158 signaling characterization of polyclonal serum HLA-A2-specific antibodies. **A.** FcγRIIIa signaling in a reporter cell line assay with high (n=7-8), medium (n=7) and low (n=8) IgG1 fucose content, and controls (n=18) in three independent assay replicates. ADCC assay performed with direct antigen (left and middle), and neutravidin-antigen (right) coated on the plate. Statistical analysis was performed using Ordinary one-way ANOVA adjusted for multiple comparisons using Tukey's test (**p*<0.05). **B.** Correlations between FcγRIIIa signaling to FcγRIIIa binding (middle panel) and HLA-A2 specific IgG MFI (lower panel) (n=30-31) across Fc signaling assay replicates. Spearman rank correlations ($R_S$) are shown in inset. **C-F.** HLA-A2-specific antibody characteristics in individuals with and without AMR. Violin plots showing HLA-A2-specific IgG MFI in individuals with AMR (n=13) versus no AMR (n=13) **(C)**, FcγRIIIa binding characterization in individuals with AMR (n=13) versus no AMR (n=13) **(D)**, FcγRIIIa signaling characterization in individuals with AMR (n=12) versus no AMR (n=13) **(E)** and FcγRIIIa dissociation rate in individuals with AMR (n=8) versus no AMR (n=2) **(F)**. Patients with and without AMR are shown in red and green, respectively. Statistical analysis was performed using a Mann-Whitney U test.

**Supplemental Table 1. Cohort characteristics, Related to Figure 1.** The median and P25-P75 are shown, unless indicated otherwise.

| Characteristics | Anti-HLA-A2 sensitized group (n=32) | Control group (n=18) |
|---|---|---|
| **Patient age - Years (P25-P75)** | 45.5 (28-57.5) | 48 (41-61) |
| **Patient gender - n (%)** | | |
| Male | 12 (37.5%) | 14 (77.7%) |
| Female | 20 (62.5) | 4 (22.2%) |
| **Sensitizing event – n (%)** | | |
| Transplantation | 13 (40.6%) | 5 (27,8%) |
| Pregnancies | 6 (18.8%) | 2 (11,1%) |
| Blood derived product transfusions | 1 (3.1%) | 1 (5,6%) |
| Left ventricular assistance device | 1 (3.1%) | 0 (0%) |
| Unknown | 11 (34.4 %) | 2 (11,1%) |
| No previous sensitizing event | 0 (0%) | 8 (44,4%) |
| **cPRA% at the time of sample collection (P25-P75)** | 95.5 (81.8-99) | 15.85(0-80.31) |
| **Type of transplant among transplanted patients – n (%)** | | |
| KT | 25 (78.1%) | 15 (83.3%) |
| KPT | 1 (3.1%) | 0 (0%) |
| HT | 1 (3.1%) | 0 (0%) |
| PT | 1 (3.1%) | 0 (0%) |
| PLT | 0 (0%) | 1 (5.5%) |
| LKT | 0 (0%) | 1 (5.5%) |
| On transplant waiting list | 4 (12.5%) | 1 (5.5%) |
| **Donor gender – n (%)** | | |
| Male | 10 (35.7%) | 13 (76,5%) |
| Female | 16 (57.1 %) | 3 (17,6%) |
| Unknown | 2 (7.1%) | 1 (5,9%) |
| **Donor age among transplanted patients (P25-P75)** * | 44 (35-54) | 40.5 (31.75-50) |
| **Donor category among transplanted patients – n (%)** | | |
| Deceased | 22 (78.56%) | 15 (88.2%) |
| Living | 3 (10.7%) | 2 (11.8%) |
| Not reported | 3 (10.7%) | 0 (0%) |
| **Warm ischemia time among transplanted patients – minutes (P25-P75)** * | 32 (27.5-38.5) | 36.5 (24.5-43.25) |
| **Cold ischemia time among transplanted patients – hours (P25-P75)** * | 19 (13-21.5) | 20 (6.95-20.5) |
| **Recovery of graft function among transplanted patients – n (%)** | | |
| Immediate | 20 (71.4%) | 14 (82,4%) |
| Delayed | 1 (3.6%) | 2 (11.8%) |
| Not reported | 7 (25%) | 1 (5,9%) |
| **HLA A-B-DR mismatches among transplanted patients – n (P25-P75)** * | 3 (2-3.5) | 3 (2-4) |
| **Induction immunosuppressive therapy among transplanted patients – n (%)** | | |
| No induction | 3 (10.7%) | 2 (11.8%) |
| Basiliximab | 6 (21.4%) | 10 (58.8%) |
| Thymoglobulin | 11 (39.3%) | 3 (17.6%) |
| OKT3 | 1 (3.6%) | 1 (5.9%) |
| Unknown | 7 (25%) | 1 (5.9%) |
| **Maintenance immunosuppressive therapy among transplanted patients – n (%)** | | |
| CNI, MMF, mPDN | 19 (67.8%) | 14 (82.4%) |
| CNI, AZA, mPDN | 3 (10.7%) | 0 (0%) |
| Others | 0 (0%) | 2 (11.8%) |
| Not reported | 6 (21.4%) | 1 (5.9%) |

| | | |
|---|---|---|
| **AMR – n (%)**<br>Unknown<br>No AMR<br>AMR<br>- Acute AMR<br>- Chronic AMR | <br>2 (7.1%)<br>13 (46.4%)<br>13 (46.4%)<br>3 (23.8%)<br>10 (76.9%) | <br>2 (11.8%)<br>12 (70.6%)<br>3 (17.6%)<br>2 (66.7%)<br>1 (33.3%) |
| **Anti-HLA-A2 fucosylation - n (%)**<br>Low<br>Medium<br>High<br>Undetermined | <br>8 (25%)<br>8 (25%)<br>8 (25%)<br>8 (25%) | Glycans not determined |
| **Time of anti-HLA-A2 antibodies detected among patients with AMR – n (%)**<br>Pre AMR<br>Post AMR | 2 (15.4%)<br>11 (84.6%) | |

* Data are given when available.

Abbreviations: KT: Kidney transplant, KPT: combined kidney pancreas transplant, HT: heart transplant, PT: pulmonary transplant, PLT: combined pulmonary liver transplant, LKT: combined liver kidney transplant.
CNI: calcineurin inhibitors; MMF: mofetil mycophenolate; mPDN methylprednisolone; AZA: Azathioprine; AMR: antibody mediated rejection.

**Supplemental Table 2. DSA cohort characteristics, Related to Figure 4 and 5.** The median and P25-P75 are shown, unless indicated otherwise.

| **Characteristics** | **Anti-HLA-A2 DSA sub-group (n=13)** |
|---|---|
| **Patient age - Years (P25-P75)** | 30 (21-44) |
| **Patient gender - n (%)** | |
| Male | 9 (64.3%) |
| Female | 5 (35.7%) |
| **Type of transplant – n (%)** | |
| KT | 12 (92.3%) |
| KPT | 1 (7.69%) |
| PLT | 0 (0%) |
| LKT | 0 (0%) |
| On transplant waiting list | 0 (0%) |
| **Donor gender – n (%)** | |
| Male | 6 (46.1%) |
| Female | 7 (53.9%) |
| Unknown | 0 (0%) |
| **Donor age– years (P25-P75)** | 41 (22-54) |
| **Donor category – n (%)** | |
| Deceased | 11 (84.6%) |
| Living | 1 (7.7%) |
| Not reported | 1 (7.7%) |
| **Warm ischemia time – minutes (P25-P75)*** | 32 (28-33) |
| **Cold ischemia time – hours (P25-P75)*** | 20 (16-26.5) |
| **Recovery of graft function – n (%)** | |
| Immediate | 10 (76.9%) |
| Delayed | 0 (0%) |
| Not reported | 3 (23.1%) |
| **HLA A-B-DR mismatches – n (P25-P75)*** | 2 (2-3.5) |
| **cPRA% at the time of sample collection (P25-P75)** | 98.3(81.4-99.6) |
| **Induction immunosuppressive therapy –n (%)** | |
| No induction | 1 (7.7%) |
| Basiliximab | 2 (15.4%) |
| Thymoglobulin | 5 (38.5%) |
| OKT3 | 1 (7.7%) |
| Not reported | 4 (30.7%) |
| **Maintenance immunosuppressive therapy – n (%)** | |
| CNI, MMF, mPDN | 6 (46.2%) |
| CNI, AZA, mPDN | 3 (23.1%) |
| Others | 0 (0%) |
| Not reported | 4 (30.8%) |
| **AMR – n (%)** | |
| Not reported | 0 (0%) |
| No AMR | 6 (46.2%) |
| AMR | 7 (53.8%) |
| - Acute AMR | 3 (42.9%) |
| - Chronic AMR | 4 (57.1%) |
| **Time of anti-HLA-A2 antibodies detected among patients with AMR** | |
| Post AMR | 7 (100%) |

| | |
|---|---|
| **Fucosylation profile among AMR positive patients - n (%)**<br>Low<br>Medium<br>High<br>No glycan information | <br>2 (28.57%)<br>4 (57.14%)<br>0 (0%)<br>1 (14.28%) |
| **Fucosylation profile among AMR negative patients – n (%)**<br>Low<br>Medium<br>High<br>No glycan information | <br>1 (16.66%)<br>0 (0%)<br>3 (50%)<br>2 (33.33%) |

* Data are given when available.

Abbreviations: KT: Kidney transplant, KPT: combined kidney pancreas transplant, HT: heart transplant, PT: pulmonary transplant, PLT: combined pulmonary liver transplant, LKT: combined liver kidney transplant.
CNI: calcineurin inhibitors; MMF: mofetil mycophenolate; mPDN methylprednisolone; AZA: Azathioprine; AMR: antibody mediated rejection.

**Supplemental Table 3. HLA-A2-specific monoclonal antibody gene sequences, Related to Figure 1 and 3.**

| Gene sequence | |
|---|---|
| **light chain** | gatgttttgatgacccaaactccactctccctgcctgtcagtcttggagatcaagtctccatctcttgcagatctagtcagagcattgtacatagtaatggaaacacctatttagaa<br>tggtacctgcagaaaccaggccagtctccaaagctcctgatctacaaagtttccaaccgattttctggggtcccagacaggttcagtggcagtggatcagggacagatttca<br>cactcaagatcagcagagtggaggctgaggatctgggagtttattactgctttcaaggttcacatgttcctcggacgttcggtggaggcaccaagctggaaatcaaaCGG<br>GCAGATGCTGCACCAACTGTATCCATCTTCCCACCATCCAGTGAGCAGTTAACATCTGGAGGTGCCTCAGTCGTGTG<br>CTTCTTGAACAACTTCTACCCCAAAGACATCAATGTCAAGTGGAAGATTGATGGCAGTGAACGACAAAATGGCGTCCT<br>GAACAGTTGGACTGATCAGGACAGCAAAGACAGCACCTACAGCATGAGCAGCACCCTCACGTTGACCAAGGACGAG<br>TATGAACGACATAACAGCTATACCTGTGAGGCCACTCACAAGACATCAACTTCACCCATTGTCAAGAGCTTCAACAGG<br>AATGAGTGT |
| **IgG1 heavy chain** | caggtccagctgcagcagtctggacctgagctggtgaagcctggggcctcagtgaagatgtcctgcaaggcttctggctacaccttcacaagctaccatatacagtgggtg<br>aagcagaggcctggacagggacttgagtggattggatggatttatcctggagatggtagtactcagtacaatgagaagttcaagggcaagaccacactgactgcagaca<br>aatcctccagcacagcctacatgttgctcagcagcctgacctctgaggactctgcgatctatttctgtgcaagggaggggacctactatgctatggactactggggtcaagga<br>acctcagtcaccgtctcctcaGCTAAAACAACACCCCCATCAGTCTATCCACTGGCCCCTGGGTGTGGAGATACAACTGGTTCC<br>TCTGTGACTCTGGGATGCCTGGTCAAGGGCTACTTCCCTGAGTCAGTGACTGTGACTTGGAACTCTGGATCCCTGTC<br>CAGCAGTGTGCACACCTTCCCAGCTCTCCTGCAGTCTGGACTCTACACTATGAGCAGCTCAGTGACTGTCCCCTCCA<br>GCACCTGGCCAAGTCAGACCGTCACCTGCAGCGTTGCTCACCCAGCCAGCAGCACCACGGTGGACAAAAAACTTGA<br>GCCCAAATCTTGTGACAAAACTCACACATGCCCACCGTGCCCAGCACCTGAACTCCTGGGGGGACCGTCAGTCTTC<br>CTCTTCCCCCCAAAACCCAAGGACACCCTCATGATCTCCCGGACCCCTGAGGTCACATGCGTGGTGGTGGACGTGA<br>GCCACGAAGACCCTGAGGTCAAGTTCAACTGGTACGTGGACGGCGTGGAGGTGCATAATGCCAAGACAAAGCCGC<br>GGGAGGAGCAGTACAACAGCACGTACCGTGTGGTCAGCGTCCTCACCGTCCTGCACCAGGACTGGCTGAATGGCA<br>AGGAGTACAAGTGCAAGGTCTCCAACAAAGCCCTCCCAGCCCCCATCGAGAAAACCATCTCCAAAGCCAAAGGGCA<br>GCCCCGAGAACCACAGGTGTACACCCTGCCCCCATCCCGGGAGGAGATGACCAAGAACCAGGTCAGCCTGACCTG<br>CCTGGTCAAAGGCTTCTATCCCAGCGACATCGCCGTGGAGTGGGAGAGCAATGGGCAGCCGGAGAACAACTACAA<br>GACCACGCCTCCCGTGCTGGACTCCGACGGCTCCTTCTTCCTCTACAGCAAGCTCACCGTGGACAAGAGCAGGTGG<br>CAGCAGGGGAACGTCTTCTCATGCTCCGTGATGCATGAGGCTCTGCACAACCACTACACGCAGAAGAGCCTCTCCC<br>TGTCTCCGGGTAAA |
| **IgG2 heavy chain** | caggtccagctgcagcagtctggacctgagctggtgaagcctggggcctcagtgaagatgtcctgcaaggcttctggctacaccttcacaagctaccatatacagtgggtg<br>aagcagaggcctggacagggacttgagtggattggatggatttatcctggagatggtagtactcagtacaatgagaagttcaagggcaagaccacactgactgcagaca<br>aatcctccagcacagcctacatgttgctcagcagcctgacctctgaggactctgcgatctatttctgtgcaagggaggggacctactatgctatggactactggggtcaagga<br>acctcagtcaccgtctcctcaGCTAAAACAACACCCCCATCAGTCTATCCACTGGCCCCTGGGTGTGGAGATACAACTGGTTCC<br>TCTGTGACTCTGGGATGCCTGGTCAAGGGCTACTTCCCTGAGTCAGTGACTGTGACTTGGAACTCTGGATCCCTGTC<br>CAGCAGTGTGCACACCTTCCCAGCTCTCCTGCAGTCTGGACTCTACACTATGAGCAGCTCAGTGACTGTCCCCTCCA<br>GCACCTGGCCAAGTCAGACCGTCACCTGCAGCGTTGCTCACCCAGCCAGCAGCACCACGGTGGACAAAAAACTTGA<br>GCGCAAATGTTGTGTCGAGTGCCCACCGTGCCCAGCACCACCTGTGGCAGGACCGTCAGTCTTCCTCTTCCCCCCA<br>AAACCCAAGGACACCCTCATGATCTCCCGGACCCCTGAGGTCACGTGCGTGGTGGTGGACGTGAGCCACGAAGAC<br>CCCGAGGTCCAGTTCAACTGGTACGTGGACGGCGTGGAGGTGCATAATGCCAAGACAAAGCCACGGGAGGAGCAG<br>TTCAACAGCACGTTCCGTGTGGTCAGCGTCCTCACCGTTGTGCACCAGGACTGGCTGAACGGCAAGGAGTACAAGT<br>GCAAGGTCTCCAACAAAGGCCTCCCAGCCCCCATCGAGAAAACCATCTCCAAAACCAAAGGGCAGCCCCGAGAACC<br>ACAGGTGTACACCCTGCCCCCATCCCGGGAGGAGATGACCAAGAACCAGGTCAGCCTGACCTGCCTGGTCAAAGG<br>CTTCTACCCCAGCGACATCGCCGTGGAGTGGGAGAGCAATGGGCAGCCGGAGAACAACTACAAGACCACGCCTCC<br>CATGCTGGACTCCGACGGCTCCTTCTTCCTCTACAGCAAGCTCACCGTGGACAAGAGCAGGTGGCAGCAGGGGAAC<br>GTCTTCTCATGCTCCGTGATGCATGAGGCTCTGCACAACCACTACACGCAGAAGAGCCTCTCCCTGTCTCCGGGTAA<br>A |
| **IgG3 heavy chain** | caggtccagctgcagcagtctggacctgagctggtgaagcctggggcctcagtgaagatgtcctgcaaggcttctggctacaccttcacaagctaccatatacagtgggtg<br>aagcagaggcctggacagggacttgagtggattggatggatttatcctggagatggtagtactcagtacaatgagaagttcaagggcaagaccacactgactgcagaca<br>aatcctccagcacagcctacatgttgctcagcagcctgacctctgaggactctgcgatctatttctgtgcaagggaggggacctactatgctatggactactggggtcaagga<br>acctcagtcaccgtctcctcaGCTAAAACAACACCCCCATCAGTCTATCCACTGGCCCCTGGGTGTGGAGATACAACTGGTTCC<br>TCTGTGACTCTGGGATGCCTGGTCAAGGGCTACTTCCCTGAGTCAGTGACTGTGACTTGGAACTCTGGATCCCTGTC<br>CAGCAGTGTGCACACCTTCCCAGCTCTCCTGCAGTCTGGACTCTACACTATGAGCAGCTCAGTGACTGTCCCCTCCA<br>GCACCTGGCCAAGTCAGACCGTCACCTGCAGCGTTGCTCACCCAGCCAGCAGCACCACGGTGGACAAAAAACTTGA<br>ATTGAAGACACCTCTCGGCGACACTACTCACACATGCCCAAGATGCCCAGAGCCTAAGTCCTGCGACACCCCTCCTC<br>CCTGTCCTAGATGCCCTGAGCCAAAGTCTTGCGATACGCCTCCACCCTGCCCTCGGTGTCCTGAGCCTAAATCATGC<br>GATACCCCACCACCATGTCCTCGCTGCCCCGCACCTGAACTCCTGGGAGGACCGTCAGTCTTCCTCTTCCCCCCAA<br>AACCCAAGGATACCCTTATGATTTCCCGGACCCCTGAGGTCACGTGCGTGGTGGTGGACGTGAGCCACGAAGACCC<br>CGAGGTCCAGTTCAAGTGGTACGTGGACGGCGTGGAGGTGCATAATGCCAAGACAAAGCCGCGGGAGGAGCAGTA<br>CAACAGCACGTTCCGTGTGGTCAGCGTCCTCACCGTCCTGCACCAGGACTGGCTGAACGGCAAGGAGTACAAGTGC<br>AAGGTCTCCAACAAAGCCCTCCCAGCCCCCATCGAGAAAACCATCTCCAAAACCAAAGGACAGCCCCGAGAACCAC<br>AGGTGTACACCCTGCCCCCATCCCGGGAGGAGATGACCAAGAACCAGGTCAGCCTGACCTGCCTGGTCAAAGGCTT<br>CTACCCCAGCGACATCGCCGTGGAGTGGGAGAGCAGCGGGCAGCCGGAGAACAACTACAACACCACGCCTCCCAT<br>GCTGGACTCCGACGGCTCCTTCTTCCTCTACAGCAAGCTCACCGTGGACAAGAGCAGGTGGCAGCAGGGGAACATC<br>TTCTCATGCTCCGTGATGCATGAGGCTCTGCACAACCGCTTCACGCAGAAGAGCCTCTCCCTGTCTCCGGGTAAA |

| | |
|---|---|
| **IgG4 heavy chain** | caggtccagctgcagcagtctggacctgagctggtgaagcctggggcctcagtgaagatgtcctgcaaggcttctggctacaccttcacaagctaccatatacagtgggtg<br>aagcagaggcctggacagggacttgagtggattggatggatttatcctggagatggtagtactcagtacaatgagaagttcaagggcaagaccacactgactgcagaca<br>aatcctccagcacagcctacatgttgctcagcagcctgacctctgaggactctgcgatctatttctgtgcaagggaggggacctactatgctatggactactggggtcaagga<br>acctcagtcaccgtctcctcaGCTAAAACAACACCCCCATCAGTCTATCCACTGGCCCCTGGGTGTGGAGATACAACTGGTTCC<br>TCTGTGACTCTGGGATGCCTGGTCAAGGGCTACTTCCCTGAGTCAGTGACTGTGACTTGGAACTCTGGATCCCTGTC<br>CAGCAGTGTGCACACCTTCCCAGCTCTCCTGCAGTCTGGACTCTACACTATGAGCAGCTCAGTGACTGTCCCCTCCA<br>GCACCTGGCCAAGTCAGACCGTCACCTGCAGCGTTGCTCACCCAGCCAGCAGCACCACGGTGGACAAAAAACTTGA<br>GTCCAAATATGGTCCCCCATGCCCATCATGCCC*A*GCACCTGAGTTCCTGGGGGGACCATCAGTCTTCCTGTTCCCCC<br>CAAAACCCAAGGACACTCTCATGATCTCCCGGACCCCTGAGGTCACGTGCGTGGTGGTGGACGTGAGCCAGGAAGA<br>CCCCGAGGTCCAGTTCAACTGGTACGTGGATGGCGTGGAGGTGCATAATGCCAAGACAAAGCCGCGGGAGGAGCA<br>GTTCAACAGCACGTACCGTGTGGTCAGCGTCCTCACCGTCCTGCACCAGGACTGGCTGAACGGCAAGGAGTACAAG<br>TGCAAGGTCTCCAACAAAGGCCTCCCGTCCTCCATCGAGAAAACCATCTCCAAAGCCAAAGGGCAGCCCCGAGAGC<br>CACAGGTGTACACCCTGCCCCCATCCCAGGAGGAGATGACCAAGAACCAGGTCAGCCTGACCTGCCTGGTCAAAGG<br>CTTCTACCCCAGCGACATCGCCGTGGAGTGGGAGAGCAATGGGCAGCCGGAGAACAACTACAAGACCACGCCTCC<br>CGTGCTGGACTCCGACGGCTCCTTCTTCCTCTACAGCAGGCTCACCGTGGACAAGAGCAGGTGGCAGGAGGGGAA<br>TGTCTTCTCATGCTCCGTGATGCATGAGGCTCTGCACAACCACTACACACAGAAGAGCCTCTCCCTGTCTCCGGGTA<br>AA |

Lowercase: $V_L$ and $V_H$
Underlined: hinge region
Red: Murine origin
Black: Human part origin

**Supplemental Table 4. Details of HLA molecules used for the purification of HLA-specific IgGs, Related to Figure 2.** Purifications using both beadsets showed comparable MFI values, ensuring that the binding of IgGs was specific to the HLA molecules and not to the loaded peptides. Purifications of all the cohort samples were done using the monomers in bold.

| Specificity | Allele | Loaded peptide | Origin |
|---|---|---|---|
| HLA-A1 | HLA A*01:01 | **VTEHDTLLY** | CMV, pp50 |
| | | CTELKLSDY | Influenza A virus, Nucleoprotein, 44-52 |
| HLA-A2 | HLA A*02:01 | **CLGGLLTMV** | EBV membrane protein, 42-434 |
| | | GLCTLVAML | EBV, mRNA export factor ICP27, 300-308 |
